# Supplementary material for: Direct visualization of transcription-replication conflicts reveals post-replicative DNA:RNA hybrids
Source: Nat Struct Mol Biol. 2023 Mar 2;30(3):348–59. doi: 10.1038/s41594-023-00928-6 (PMC10023573; doi:10.1038/s41594-023-00928-6)
Supplement: Supplementary file 1 — Supplementary Table: key materials. [file 41594_2023_928_MOESM1_ESM.pdf]

nature structural & molecular biology

Article

https://doi.org/10.1038/s41594-023-00928-6

Direct visualization of transcription-replication conflicts reveals post-replicative DNA:RNA hybrids

In the format provided by the authors and unedited

nature structural & molecular biology

Article

https://doi.org/10.1038/s41594-023-00928-6

Direct visualization of transcription-replication conflicts reveals post-replicative DNA:RNA hybrids

In the format provided by the authors and unedited

Supplementary table: Key materials

REAGENT or RESOURCE	SOURCE	IDENTIFIER
Antibodies		
Mouse S9.6 DNA:RNA hybrid	Kerafast	Cat # ENH001; AB_2687463
Mouse S9.6-Gold NPS conjugate	BSI	This paper
Mouse anti-dsDNA [HYB331-01]	Abcam	Cat # ab27156; AB_470907
Rat anti-BrdU (CldU) [BU1/75 (ICR1)]	Abcam	Cat # ab6326; AB_305426
Mouse anti-BrdU (IdU)	Becton Dickinson	Cat # 347580; AB_10015219
Mouse anti-gH2AX (Ser139), JBW301	Millipore	Cat # 05-636; AB_309864
Rabbit anti-ZRANB3	Proteintech	Cat # 23111-1-AP; AB_2744527
Mouse anti-actin, AC-15	Sigma-Aldrich	Cat # A5441; AB_476744
Goat anti-mouse-AlexaFluor488	Thermo Fisher Scientific	Cat # A-11001; AB_2534069
Goat anti-mouse-AlexaFluor 647	Thermo Fisher Scientific	Cat # A-21235; AB_2535804
Donkey anti-rat-Cy3	LubioScience	Cat # 712-166-153; AB_2340669
Anti-rabbit-HRP linked	VWR	Cat # NA934; AB_772206
Anti-mouse-HRP linked	VWR	Cat # NA931; AB_772210
Bacterial and Virus Strains		
B. subtilis phe trp amyE::$P_{spank(hy)}$-*lacZ* (HO)	Lang et al 2017	HM1300
B. subtilis phe trp Δ*rnhC amyE*::$P_{spank(hy)}$-*lacZ* (HO)	Lang et al 2017	HM2043
Chemicals, peptides, and recombinant proteins		
Low melting NuSieve GTG agarose	Avantor VWR	Cat # 50081
SeaPlaque low-melting point agarose	Lonza	Cat # 50,111
Ultrapure agarose	Thermo Fisher Scientific	Cat # 16500100
Alexa Fluor 647 Azide	Thermo Fisher Scientific	Cat # A10277
SYBR Gold Nucleic Acid Gel Stain	Thermo Fisher Scientific	Cat # S11494
Ethidium bromide	Sigma-Aldrich	Cat # E1510
Benzyldimethylalkyl Ammonium Chloride	Sigma-Aldrich	Cat # B6295
Formamide	Sigma-Aldrich	Cat # 47680
Glutaraldehyde 25%	EMS	Cat # 16200
Uranyl acetate	Fluka	Cat # 73943
T3 RNA polymerase	Promega	Cat # P2083
RNase A	Sigma-Aldrich	Cat # R5503
RNase H	NEB	Cat # M0297L
RNase III	Thermo Fisher Scientific	Cat # AM2290
RNase T1	Thermo Fisher Scientific	Cat # EN0541
Proteinase K	Sigma-Aldrich	Cat # 3115852001
BbvCI	NEB	Cat # R0601
BsrGI	NEB	Cat # R3575
EcoRI	NEB	Cat # R3101
HindIII-HF	NEB	Cat # R3504
NotI	NEB	Cat # R3189
PvuII	NEB	Cat # R3151
SspI	NEB	Cat # R3132
XmnI	NEB	Cat # R0194
PARPi	Selleckchem	Cat # S1060
Beta-estradiol	Sigma-Aldrich	Cat # E2758
Camptothecin	Sigma-Aldrich	Cat #: C9911
Doxycycline	Sigma-Aldrich	Cat #: D9891
Light Cycler 480 SYBR Green	Roche	Cat # 4707516001
ProLong Gold Antifade Mountant	Thermo Fisher Scientific	Cat # P36930
Western Bright ECL-HRP Substrate	Advansta	Cat # K-12045
RNAiMAX	Thermo Fisher	Cat # 13778500

Lipofectamine 3000	Thermo Fisher	Cat # L3000008
Critical commercial assays		
Silica Bead DNA Gel Extraction Kit	Thermo Fisher Scientific	Cat # K0513
Click-it EdU AlexaFluor 488 Flow Cytometry Assay Kit	Thermo Fisher Scientific	Cat # C10420
Experimental models: cell lines		
Human: MCF7	Gift from Karlene Cimprich (Stanford, USA)	N/A
Human: HeLa TRIPZ control	Gift from the Pasero lab (IGH, France)	N/A
Human: Hela TRIPZ shTOP1	Gift from the Pasero lab (IGH, France)	N/A
Human: U2OS	ATCC; HTB-96	N/A
Oligonucleotides		
qPCR primer: HM188 (*B. subtilis*, conflict)	Lang et al 2017	GGCTTTCGCTACCTGGAGAG
qPCR primer: HM189 (*B. subtilis*, conflict)	Lang et al 2017	GACGAAGCCGCCCTGTAAAC
qPCR primer: HM192 (*B. subtilis*, control)	Lang et al 2017	CCGTCTGACCCGATCTTTTA
qPCR primer: HM193 (*B. subtilis*, control)	Lang et al 2017	GTCATGCTGAATGTCGTGCT
qPCR primer: 83/84_FWD (MCF7)	Stork et al 2016	GAACGTTCAGCCTCGTTCTC
qPCR primer: 83/84_REV (MCF7)	Stork et al 2016	GGAAGGTGGAAGGAAACACA
qPCR primer: GREB_1 FWD (MCF7)	Stork et al 2016	AGGCCGCGTGAGTAAAGAG
qPCR primer: GREB_1 REV (MCF7)	Stork et al 2016	TCTGGAGCCAGGAAGACTTG
qPCR primer: SLC7A5_1_FWD (MCF7)	Stork et al 2016	CCCCCACAACTTGTAAACCA
qPCR primer: SLC7A5_1_REV (MCF7)	Stork et al 2016	CGAGGGGAGAGGGGTAAG
qPCR primer: RPL13A_FWD (MCF7)	Stork et al 2016	AGGTGCCTTGCTCACAGAGT
qPCR primer: RPL13A_REV (MCF7)	Stork et al 2016	GGTTGCATTGCCCTCATTAC
siRNA: ZRANB3	Dharmacon	Cat # 84083
Recombinant DNA		
pFC53	Ginno et al 2012	N/A
pML113	Follonier et al 2013	N/A
pAIO RNH1-GFP	Gift from the Janscak lab (IMCR, Zurich)	N/A
Software and algorithms		
ImageJ (version 2.0.0-rc-43/1.51h)	National Institute of Health	N/A
Prism 8 (version 8.4.2)	GraphPad	N/A
Maps Viewer (Version 3.16)	Thermo Fisher Scientific	N/A
OpenComet plugin Version 1.3.1	OpenComet Software	N/A
FlowJo Software (version 10.4)	BD Life Sciences	N/A
ForkStitcher https://github.com/jluethi/ForkStitcher, Version 0.1.1.	Stoy et al, MiMB 2022a	N/A
DNA content quantification algorithm https://github.com/roessler-f/DNAQuantification	Stoy et al, MiMB 2022a	N/A
Other		
Charcoal stripped FBS	Thermo Fisher Scientific	Cat # 12676029
Phenol-red free DMA	Thermo Fisher Scientific	Cat # 21063045
Amicon Ultra-0.5 Centrifugal Filter Unit	Millipore	Cat # UFC510096
Zeta-Probe Blotting Membranes	Bio-rad Laboratories	Cat # 1620165
