# Supplementary material for: Direct visualization of transcription-replication conflicts reveals post-replicative DNA:RNA hybrids
Source: Nat Struct Mol Biol. 2023 Mar 2;30(3):348–59. doi: 10.1038/s41594-023-00928-6 (PMC10023573; doi:10.1038/s41594-023-00928-6)
Supplement: Supplementary file 2 — Reporting Summary [file 41594_2023_928_MOESM2_ESM.pdf]

nature portfolio

Corresponding author(s): Massimo Lopes

Last updated by author(s): Dec 22, 2022

Reporting Summary

Nature Portfolio wishes to improve the reproducibility of the work that we publish. This form provides structure for consistency and transparency in reporting. For further information on Nature Portfolio policies, see our Editorial Policies and the Editorial Policy Checklist.

Statistics

For all statistical analyses, confirm that the following items are present in the figure legend, table legend, main text, or Methods section.

n/a	Confirmed	
☐	☒	The exact sample size (*n*) for each experimental group/condition, given as a discrete number and unit of measurement
☐	☒	A statement on whether measurements were taken from distinct samples or whether the same sample was measured repeatedly
☐	☒	The statistical test(s) used AND whether they are one- or two-sided<br>*Only common tests should be described solely by name; describe more complex techniques in the Methods section.*
☒	☐	A description of all covariates tested
☐	☒	A description of any assumptions or corrections, such as tests of normality and adjustment for multiple comparisons
☐	☒	A full description of the statistical parameters including central tendency (e.g. means) or other basic estimates (e.g. regression coefficient) AND variation (e.g. standard deviation) or associated estimates of uncertainty (e.g. confidence intervals)
☐	☒	For null hypothesis testing, the test statistic (e.g. *F*, *t*, *r*) with confidence intervals, effect sizes, degrees of freedom and *P* value noted<br>*Give P values as exact values whenever suitable.*
☒	☐	For Bayesian analysis, information on the choice of priors and Markov chain Monte Carlo settings
☒	☐	For hierarchical and complex designs, identification of the appropriate level for tests and full reporting of outcomes
☒	☐	Estimates of effect sizes (e.g. Cohen's *d*, Pearson's *r*), indicating how they were calculated

Our web collection on statistics for biologists contains articles on many of the points above.

Software and code

Policy information about availability of computer code

Data collection	Dot blot/Immunoblot: Fusion-Capt Advance Solo7 (Vilber Lourmat); Electron microscopy: DigitalMicrograph Version 1.83.842 (Gatan, Inc.) or MAPS Version 3.16 (Thermo Fisher Scientific); DNA fiber imaging: Leica Application Suite X 3.6.0.20104; Flow cytometry: Attune NXT Software Version 4.2.0
Data analysis	Dot blot, DNA fibers and Electron Microscopy (EM): ImageJ Version 2.0.0-rc-43/1.51h; Automated EM: MAPS Viewer Version 3.16; fork stitcher (https://github.com/jluethi/ForkStitcher, Version 0.1.1) and DNA content quantification algorithm (https://github.com/roessler-f/DNAQuantification); Flow cytometry: FlowJo Version 10.4; Olive tail moment: Open Comet plugin (version 1.3.1) for ImageJ; statistical analysis: Prism Version 8.4.2.

For manuscripts utilizing custom algorithms or software that are central to the research but not yet described in published literature, software must be made available to editors and reviewers. We strongly encourage code deposition in a community repository (e.g. GitHub). See the Nature Portfolio guidelines for submitting code & software for further information.

Data

Policy information about availability of data

All manuscripts must include a data availability statement. This statement should provide the following information, where applicable:

- Accession codes, unique identifiers, or web links for publicly available datasets
- A description of any restrictions on data availability
- For clinical datasets or third party data, please ensure that the statement adheres to our policy

Source data underlying all figures are provided in the source data file. Uncropped, original microscopy images and blots underlying all figures are provided as PDFs. Remaining original microscopy image data sets exceed several terabytes and will be made available upon reasonable request. The code for fork stitching is available

at: https://github.com/jluethi/ForkStitcher, Version 0.1.1. The code for the DNA content quantification algorithm can be found at: https://github.com/roessler-f/DNAQuantification.

Field-specific reporting

Please select the one below that is the best fit for your research. If you are not sure, read the appropriate sections before making your selection.

☒ Life sciences ☐ Behavioural & social sciences ☐ Ecological, evolutionary & environmental sciences

For a reference copy of the document with all sections, see nature.com/documents/nr-reporting-summary-flat.pdf

Life sciences study design

All studies must disclose on these points even when the disclosure is negative.

Sample size	Sample size for all experiments shown (electron microscopy, n>70 in 2 or more independent experiments; DNA fibers, n>100 in 2 or more independent experiments) was chosen to obtain statistical power, in conformity to accepted standard sample size in a number of previous publications using these approaches:<br><br>Mijic et al., Nat Commun., DOI: 10.1038/s41467-017-01164-5<br>Vujanovic et al., Mol Cell, DOI: 10.1016/j.molcel.2017.08.010<br>Mutreja et al., Cell Rep., DOI: 10.1016/j.celrep.2018.08.019
Data exclusions	N/A
Replication	For all experiments, the number of biological replicates is indicated in the figure legends and, without any exceptions, representative data shown in the figures was reproduced at least once.
Randomization	N/A
Blinding	Investigators were blinded for the data analysis of individual DNA fiber and Electron microscopy experiments. Blinding of the experimenter during data acquisition was not needed as most pipelines are automated, hence intrinsically unbiased.

Reporting for specific materials, systems and methods

We require information from authors about some types of materials, experimental systems and methods used in many studies. Here, indicate whether each material, system or method listed is relevant to your study. If you are not sure if a list item applies to your research, read the appropriate section before selecting a response.

Materials & experimental systems

n/a	Involved in the study	
☐	☒	Antibodies
☐	☒	Eukaryotic cell lines
☒	☐	Palaeontology and archaeology
☒	☐	Animals and other organisms
☒	☐	Human research participants
☒	☐	Clinical data
☒	☐	Dual use research of concern

Methods

n/a	Involved in the study	
☒	☐	ChIP-seq
☒	☐	Flow cytometry
☒	☐	MRI-based neuroimaging

Antibodies

Antibodies used	Primary antibodies:<br>Mouse S9.6 DNA:RNA hybrid, Kerafast, Cat # ENH001; AB_2687463;<br>Mouse S9.6-Gold NPS conjugate, BSI, this paper;<br>Mouse anti-dsDNA [HYB331-01], Abcam, Cat # ab27156; AB_470907;<br>Rat anti-BrdU (CldU) [BU1/75 (ICR1)], Abcam, Cat # ab6326; AB_305426;<br>Mouse anti-BrdU (IdU), clone B44 , Becton Dickinson, Cat # 347580; AB_10015219;<br>Mouse anti-gH2AX (Ser139), JBW301, Millipore, Cat # 05-636; AB_309864;<br>Rabbit anti-ZRANB3, Proteintech, Cat # 23111-1-AP; AB_2744527;<br>Mouse anti-actin, Sigma-Aldrich, Cat # A5441; AB_476744.<br><br>Secondary antibodies:<br>Goat anti-mouse-AlexaFluor488, Thermo Fisher Scientific, Cat # A-11001; AB_2534069;<br>Goat anti-mouse-AlexaFluor 647, Thermo Fisher Scientific, Cat # A-21235; AB_2535804;<br>Donkey anti-rat-Cy3, LubioScience, Cat # 712-166-153; AB_2340669;

	Anti-rabbit-HRP linked, VWR, Cat # NA934; AB_772206;<br>Anti-mouse-HRP linked, VWR, Cat # NA931; AB_772210.
Validation	The Mouse S9.6 DNA:RNA hybrid antibody has been tested in a plethora of studies for the use in affinity binding assays, ChIP, ChIP-seq, IP, Immunocytochemistry as claimed by the provider: s://www.kerafast.com/productgroup/432/anti-dna-rna-hybrid-s96-antibody. We validated this antibody in our own hands with RNase H controls.<br><br>The S9.6-Gold conjugate was validated in this study by electron microscopy, using an in vitro assay in combination with RNase H controls (Figure 1 and Extended Data Figure 1).<br><br>For the mouse anti-dsDNA antibody, the provider (Abcam, https://www.abcam.com/ds-dna-antibody-35i9-dna-bsa-and-azide-free-ab27156.html) has validated the following specificity: Primarily Double stranded DNA. Measurements by immuno-CE yielded KD's of 0.71 µM and 0.09 µM, for the interaction of this antibody with ss- and dsDNA, respectively. Strong reactivity with both ss- and dsDNA has been observed on dotblots as well as very weak reactivity with RNA.<br><br>The rat anti-BrdU antibody was validated for ICC/IF, IHC-P, Flow Cyt as reported by Abcam (https://www.abcam.com/brdu-antibody-bu175-icr1-proliferation-marker-ab6326.html) and has been in use and published by our own lab for the last decade (e.g. Mijic et al., Nat Commun., DOI: 10.1038/s41467-017-01164-5; Vujanovic et al., Mol Cell, DOI: 10.1016/j.molcel.2017.08.010; Mutreja et al., Cell Rep., DOI: 10.1016/j.celrep.2018.08.019).<br><br>The mouse anti-BrdU (IdU) antibody has been validated by the company (BD Biesciences, https://www.bdbiosciences.com/en-ch/products/reagents/flow-cytometry-reagents/clinical-discovery-research/single-color-antibodies-ruo-gmp/purified-mouse-anti-brdu.347580) and has been used and published by our own lab for the last decade (e.g. Mijic et al., Nat Commun., DOI: 10.1038/s41467-017-01164-5; Vujanovic et al., Mol Cell, DOI: 10.1016/j.molcel.2017.08.010; Mutreja et al., Cell Rep., DOI: 10.1016/j.celrep.2018.08.019).<br><br>The mouse anti-gH2AX has been tested in Western blotting, ICC, ChIP & Immunofluorescence (https://www.merckmillipore.com/CH/de/product/Anti-phospho-Histone-H2A.X-Ser139-Antibody-clone-JBW301,MM_NF-05-636-I?ReferrerURL=https%3A%2F%2Fwww.google.com%2F#).<br><br>The rabbit anti-ZRANB3 antibody has been KO and KD validated in many publications including several from our own lab: DOI: 10.1016/j.molcel.2017.08.010; DOI: 10.1038/s41467-017-01164-5; DOI: 10.1016/j.molcel.2019.10.026 and more as stated on the vendors website: https://www.ptglab.com/products/ZRANB3-Antibody-23111-1-AP.htm.<br><br>The monoclonal anti-beta-actin antibody has been claimed as validated for IF, IHC and protein arrays by Sigma-Aldrich: https://www.sigmaaldrich.com/CH/de/product/sigma/a5441.<br><br>All secondary antibodies were validated with a control missing the primary antibody.

Eukaryotic cell lines

Policy information about cell lines

Cell line source(s)	Human MCF7 (kind gift from Karlene Cimprich, Stanford CA, USA); human HeLa TRIPZ control and human HeLa TRIPZ shTOP1 (gift from Philippe Pasero, IGH, France); U2OS cells (ATCC, HTB-96)
Authentication	None of the cell lines were authenticated in house for this manuscript.
Mycoplasma contamination	MCF7 and U2OS have been repeatedly tested negative for mycoplasma in our routine in-house tests.
Commonly misidentified lines<br>(See ICLAC register)	No commonly misidentified lines were used in this study.
