# Supplementary figures and images for: Direct visualization of transcription-replication conflicts reveals post-replicative DNA:RNA hybrids
Source: Nat Struct Mol Biol. 2023 Mar 2;30(3):348–59. doi: 10.1038/s41594-023-00928-6 (PMC10023573; doi:10.1038/s41594-023-00928-6)

Figure 1b

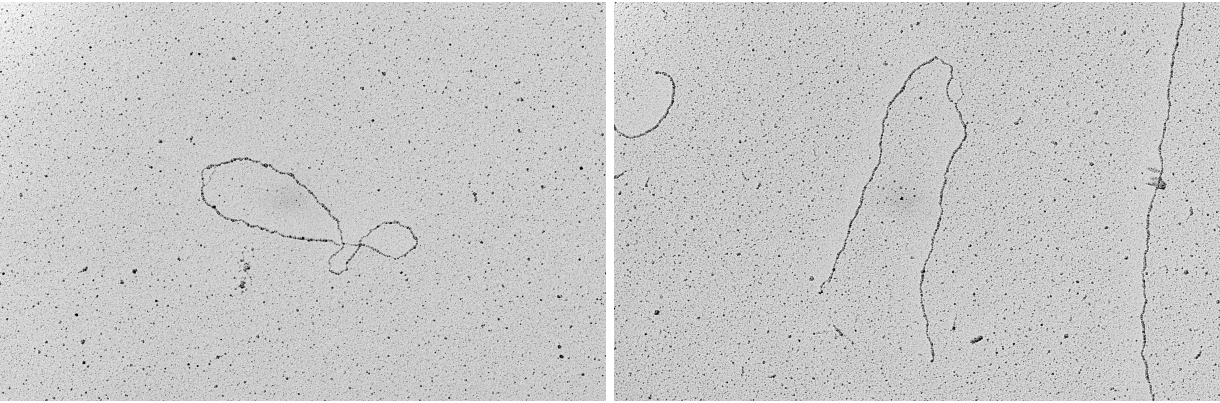

Figure 1f

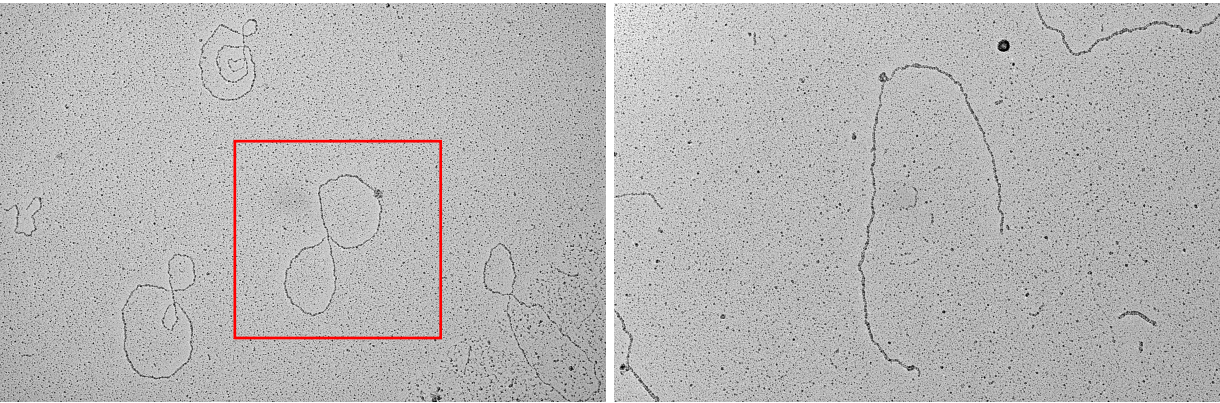

Supplement: Source Data Fig. 1 — Uncropped representative images. [file 41594_2023_928_MOESM4_ESM.pdf]

Figure 4d - I

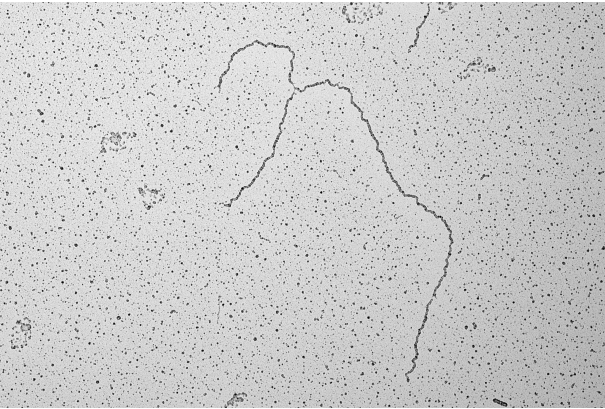

Figure 4d - II

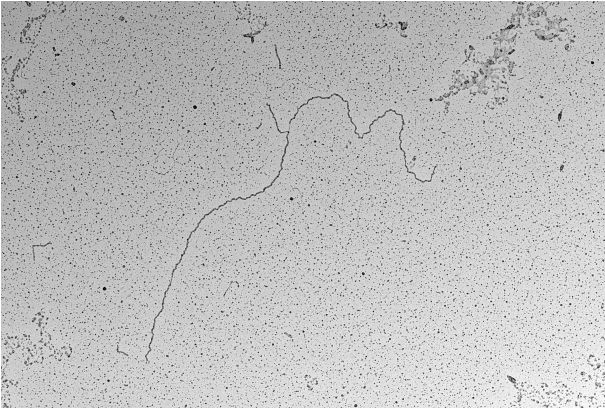

Figure 4d - III

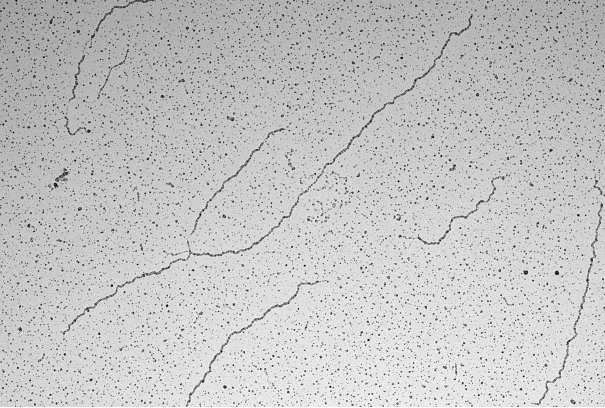

Figure 4d - IV

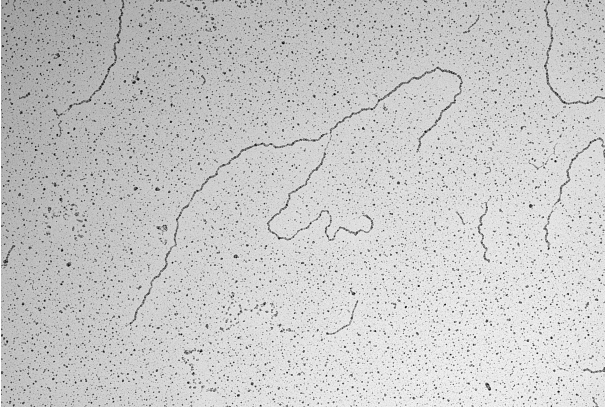

Figure 4d - V

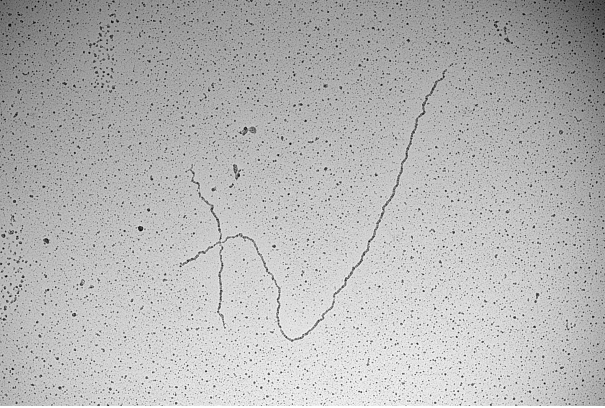

Figure 4d - VI

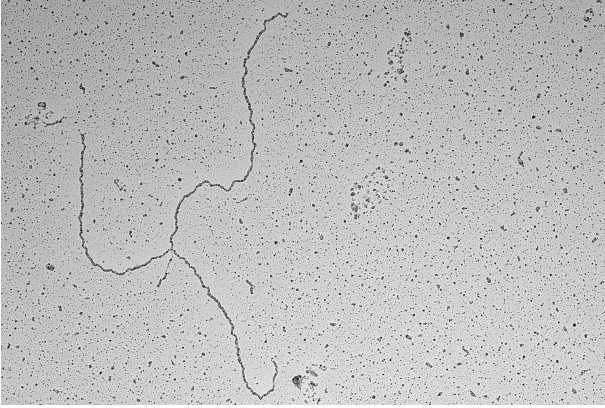

Supplement: Source Data Fig. 4 — Uncropped representative images. [file 41594_2023_928_MOESM7_ESM.pdf]

Figure 5a

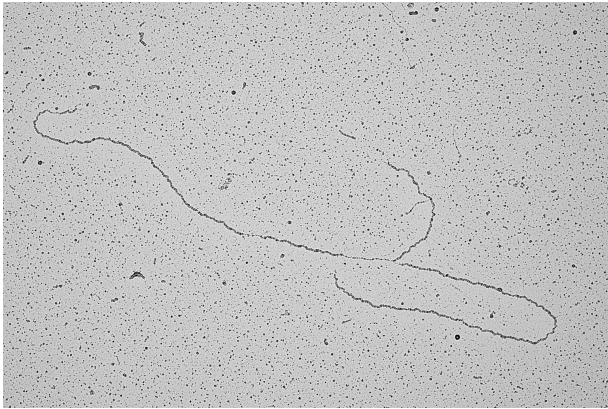

Figure 5e

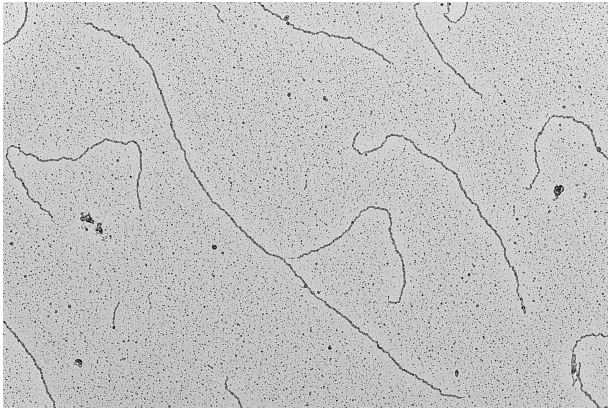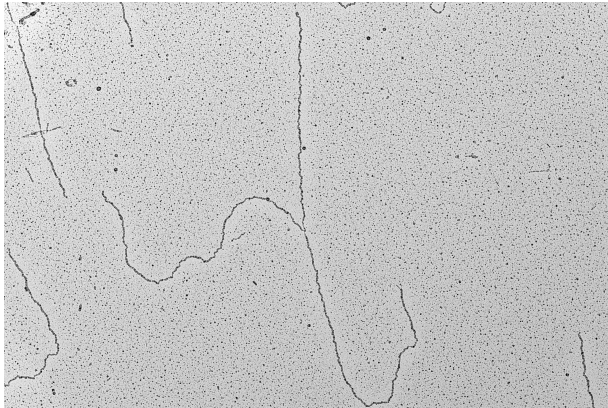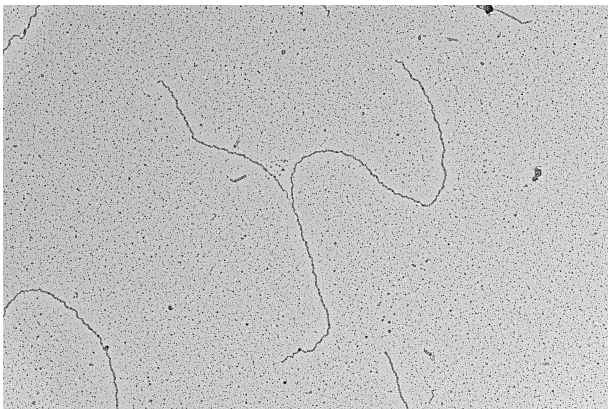

Supplement: Source Data Fig. 5 — Uncropped representative images. [file 41594_2023_928_MOESM8_ESM.pdf]

Extended Data Figure 1b

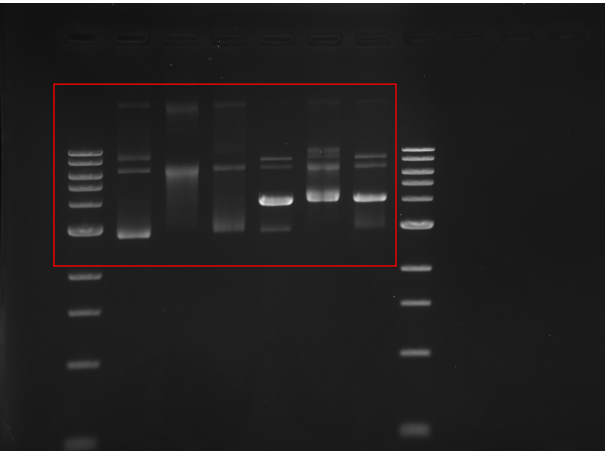

Extended Data Figure 1c

S9.6

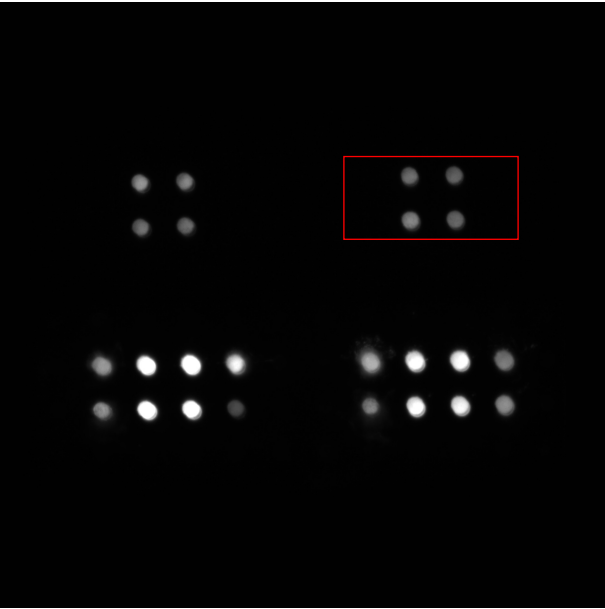

dsDNA

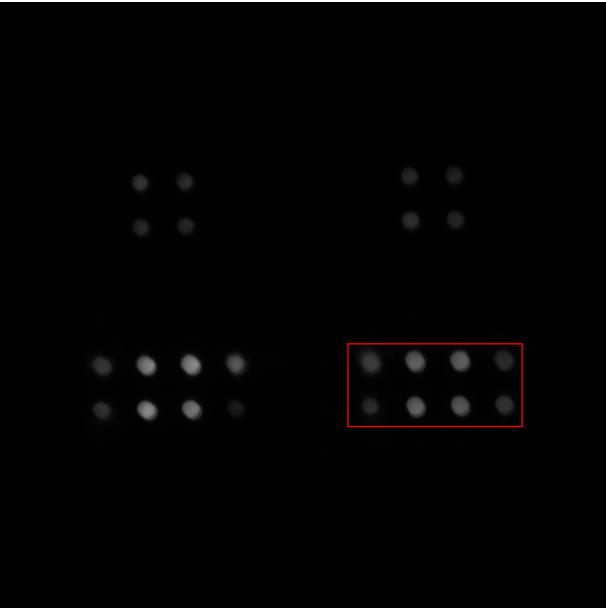

Supplement: Source Data Extended Data Fig. 1 — Unprocessed blots and gels. [file 41594_2023_928_MOESM9_ESM.pdf]

Extended Data Figure 4c

ZRANB3

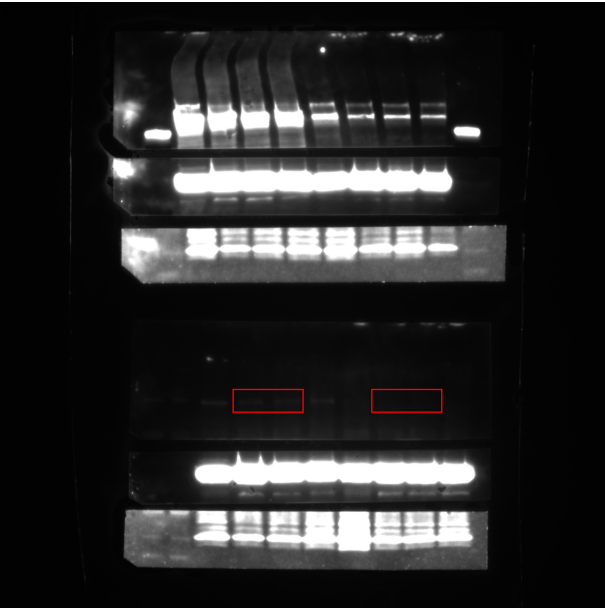

Actin

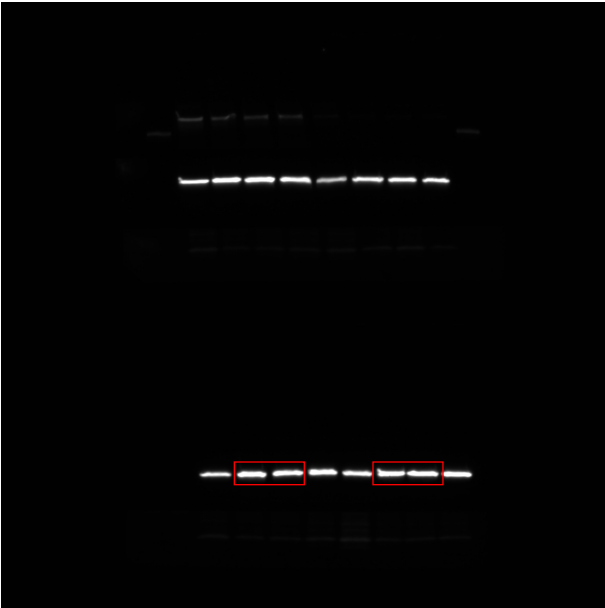

Extended Data Figure 4e - left

- E2

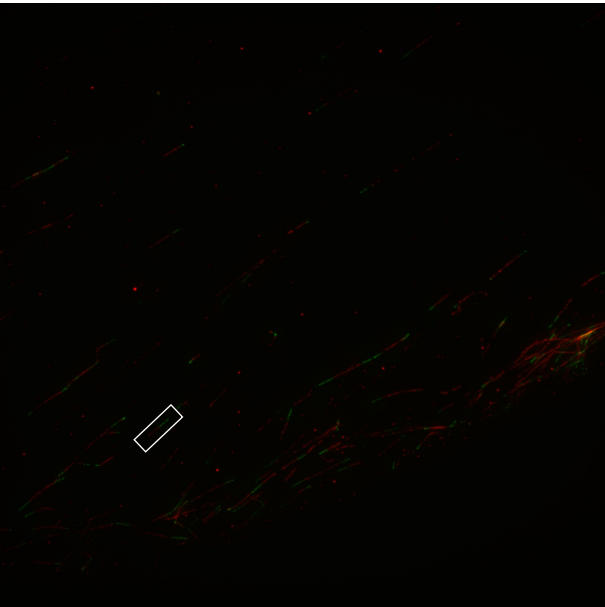

- E2 + PARPi

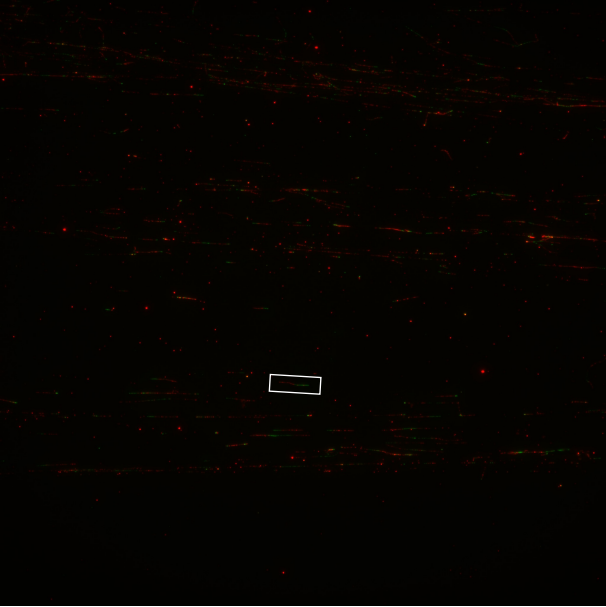

+ E2

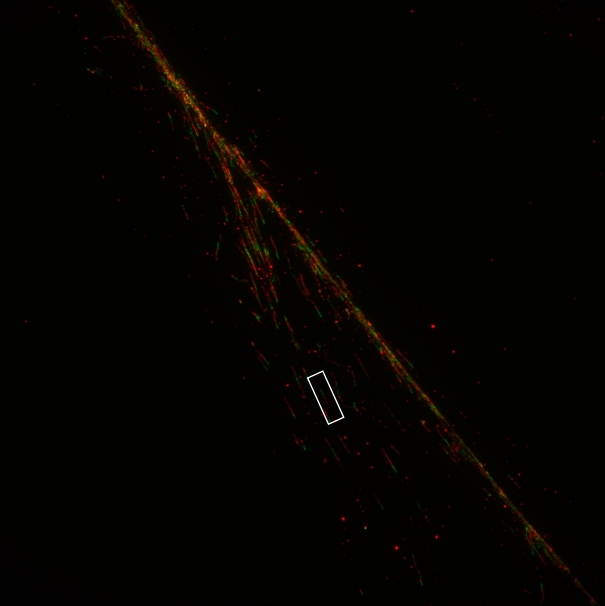

+ E2 + PARPi

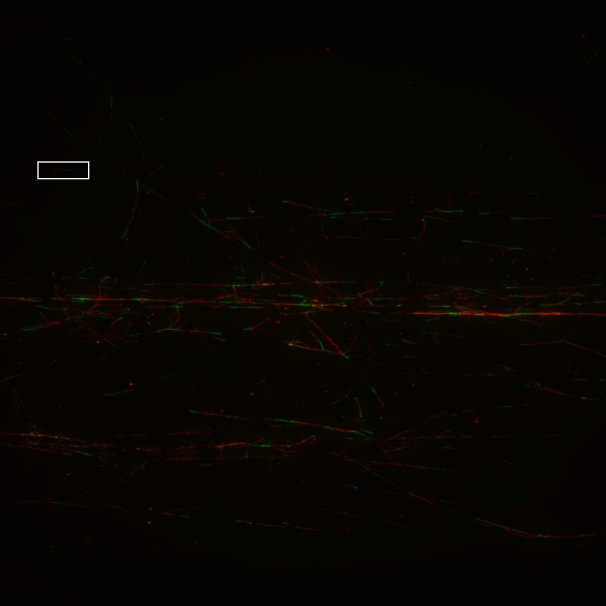

Supplement: Source Data Extended Data Fig. 4 — Unprocessed blots and representative images. [file 41594_2023_928_MOESM12_ESM.pdf]

Extended Data Figure 5a

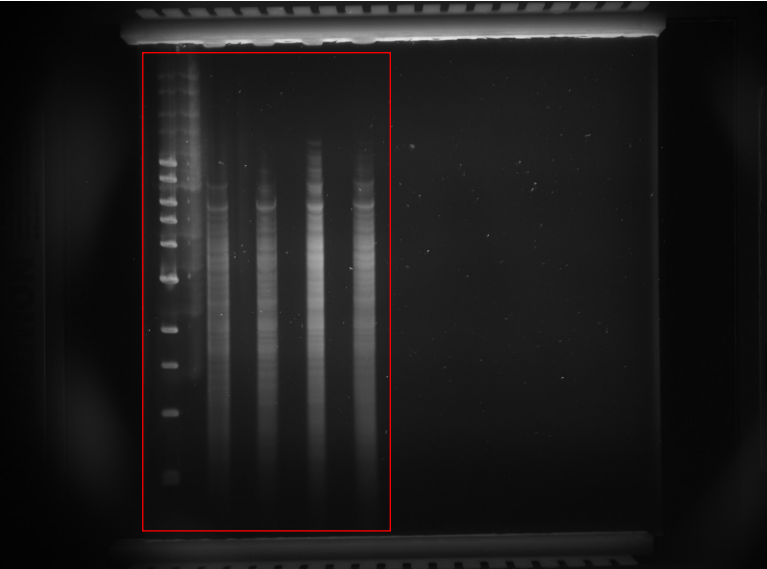

Extended Data Figure 5b

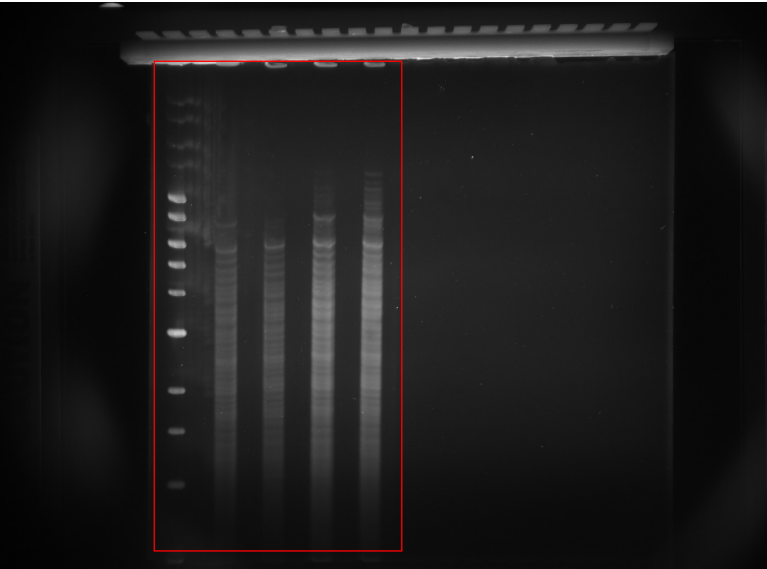

Extended Data Figure 5c

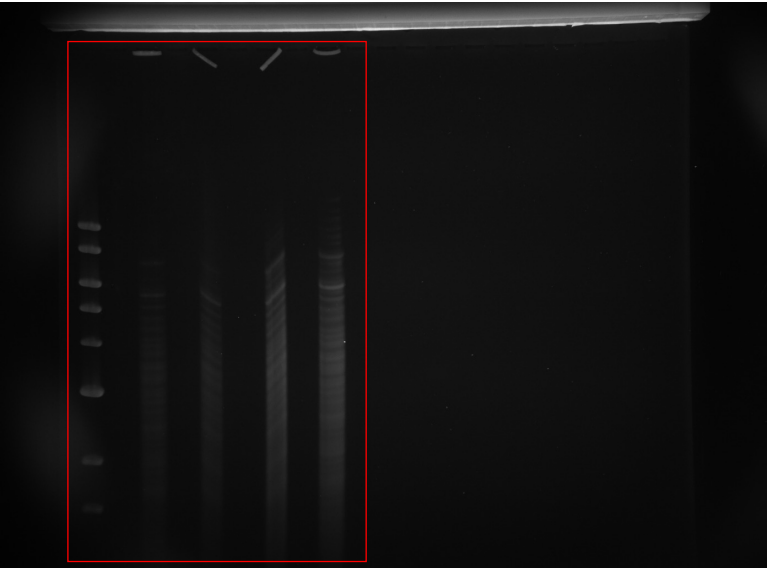

Supplement: Source Data Extended Data Fig. 5 — Unprocessed gels. [file 41594_2023_928_MOESM13_ESM.pdf]
