# Supplementary material for: Direct visualization of transcription-replication conflicts reveals post-replicative DNA:RNA hybrids
Source: Nat Struct Mol Biol. 2023 Mar 2;30(3):348–59. doi: 10.1038/s41594-023-00928-6 (PMC10023573; doi:10.1038/s41594-023-00928-6)

Figure 3a - left

- E2 - RNH1-GFP

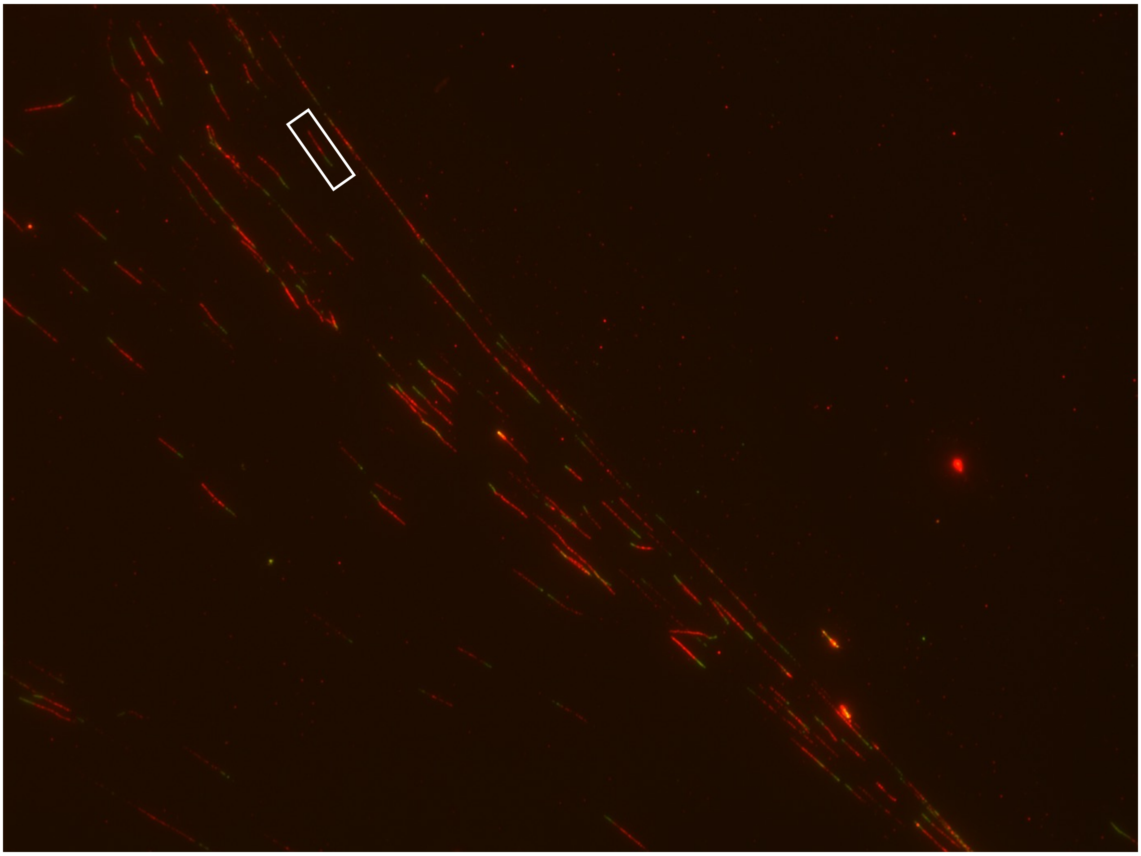

- E2 + RNH1-GFP

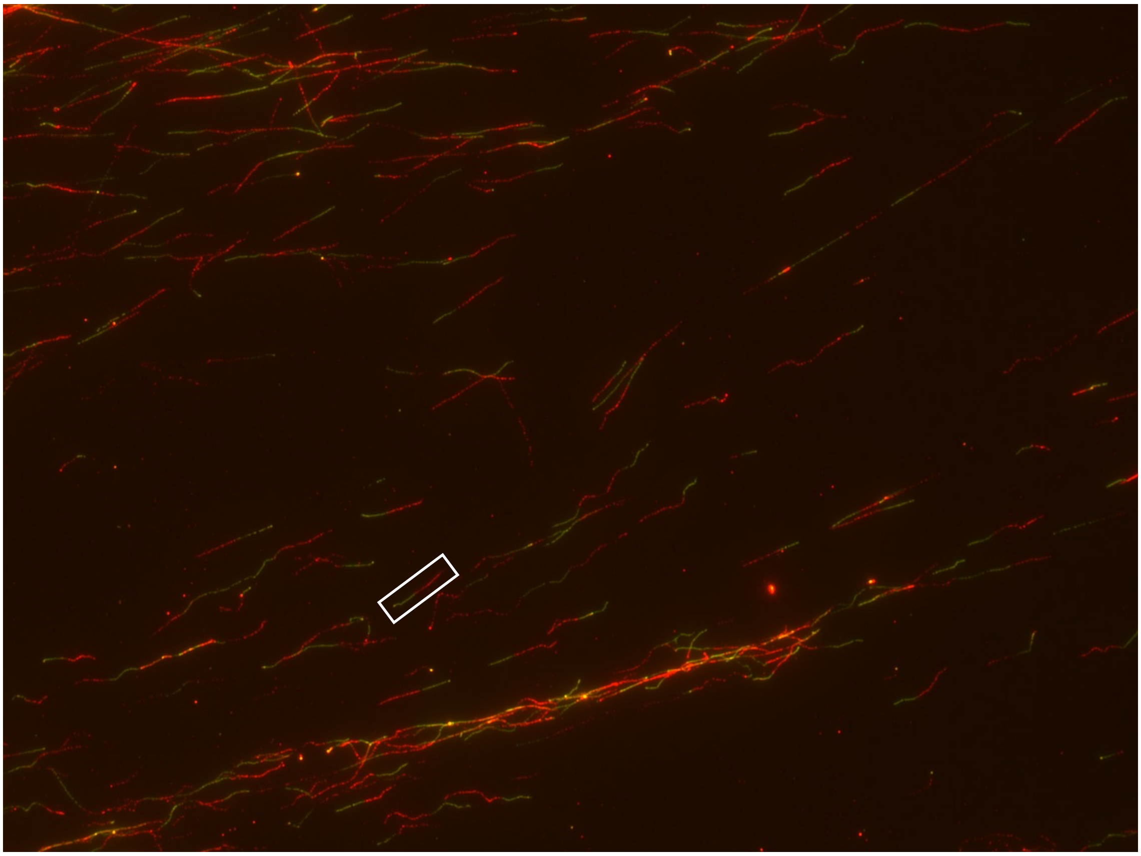

Figure 3a - left

+ E2 - RNH1-GFP

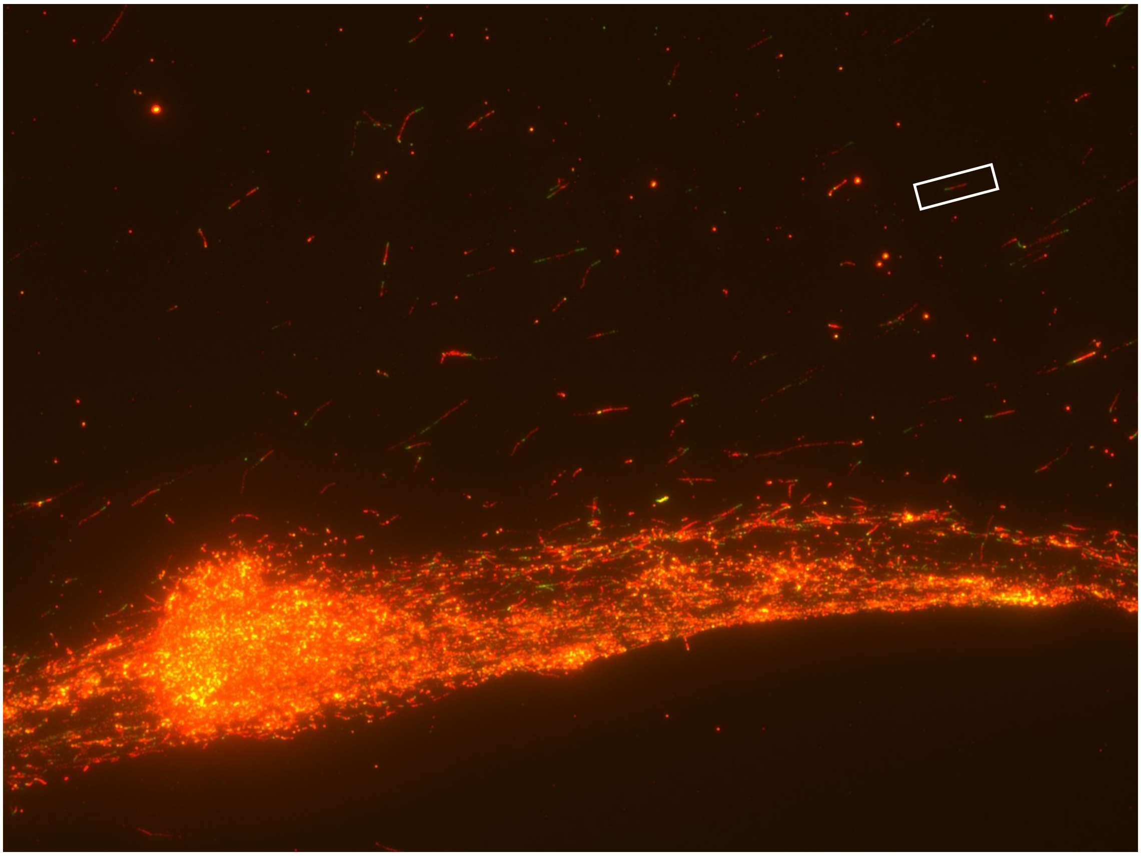

+ E2 + RNH1-GFP

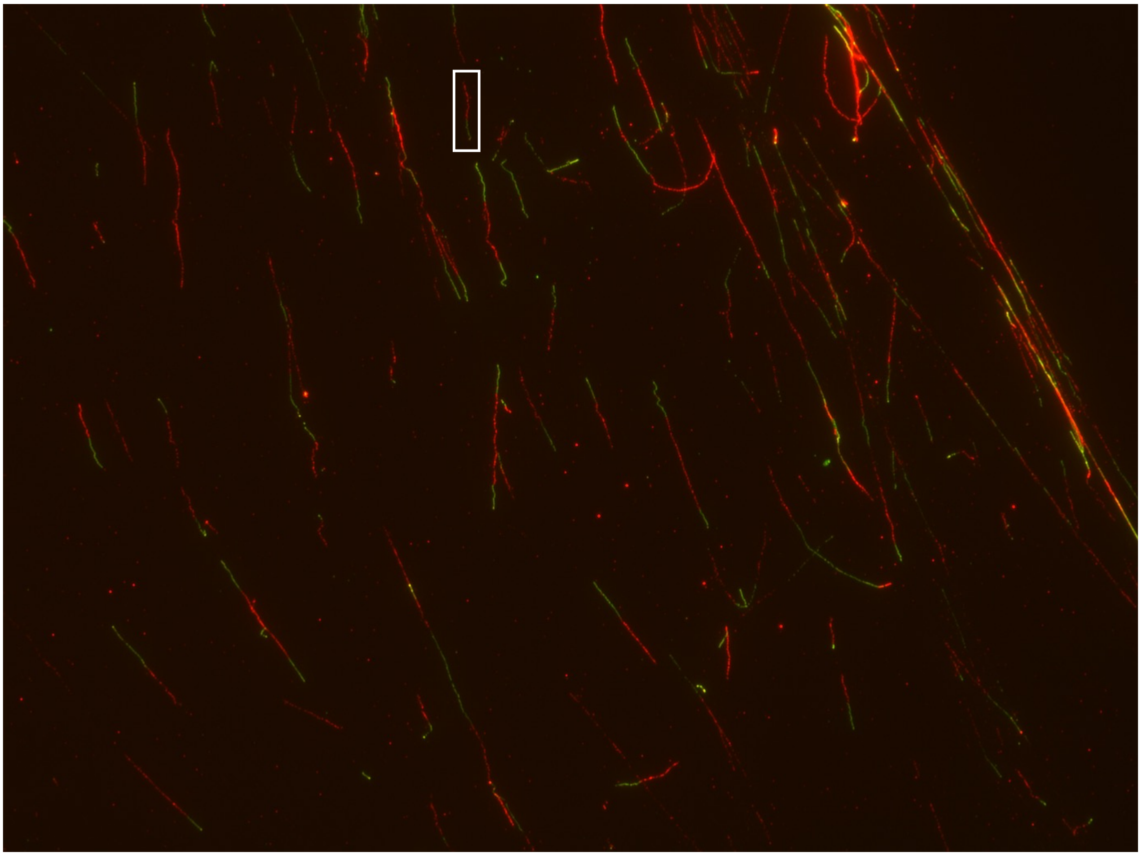

Figure 3b - left

- E2 siLuc

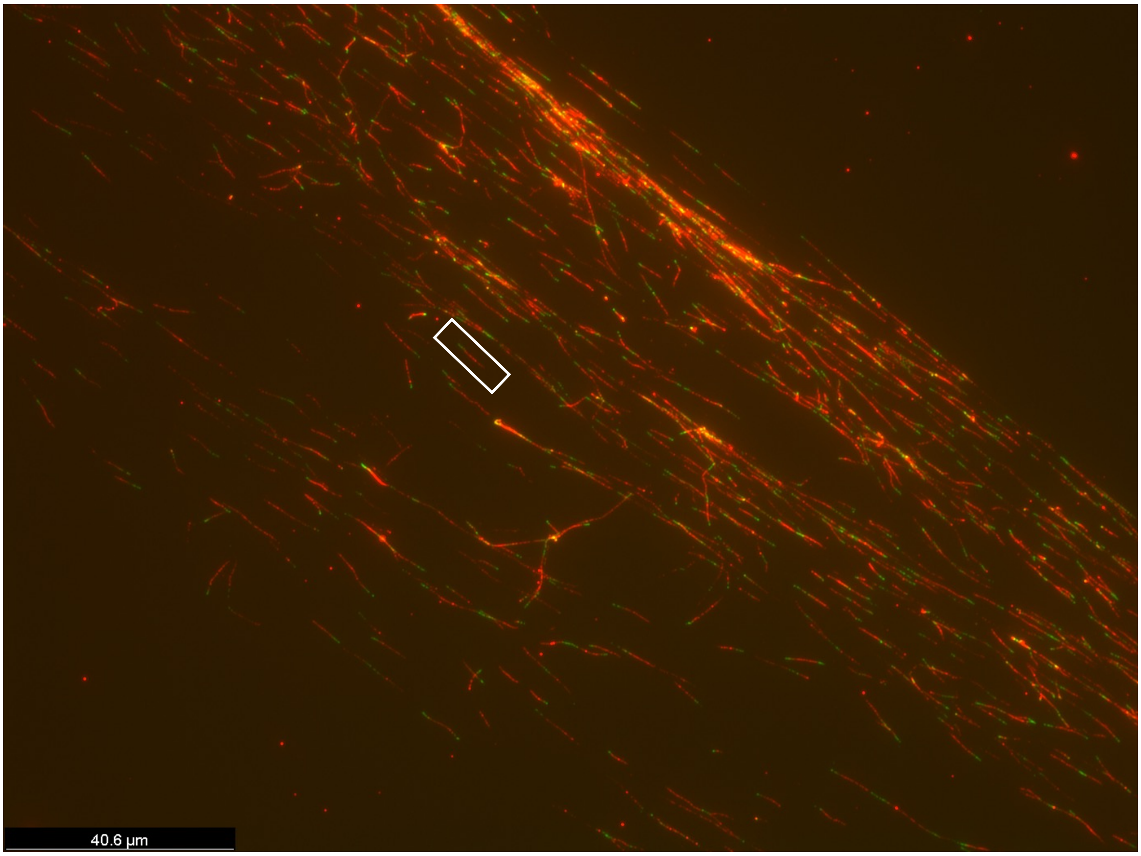

- E2 siZ3

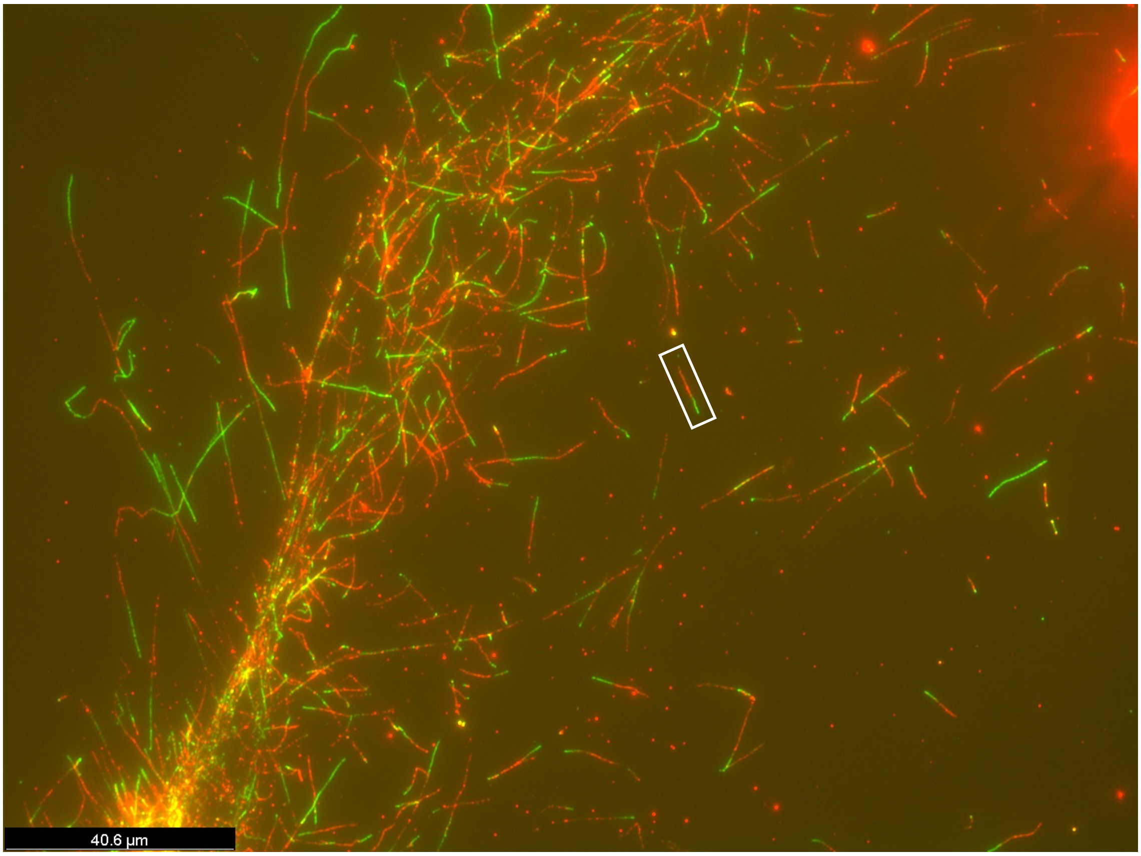

Figure 3b - left

+ E2 siLuc

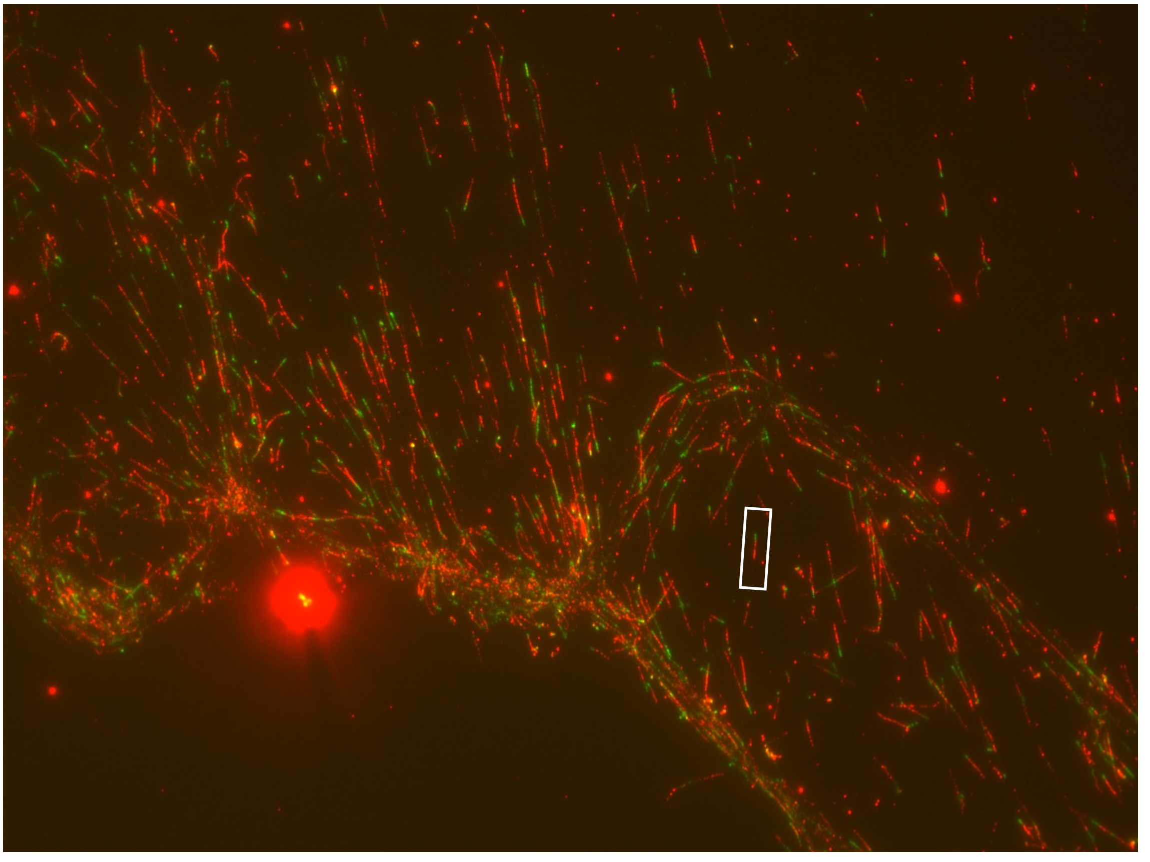

+ E2 siZ3

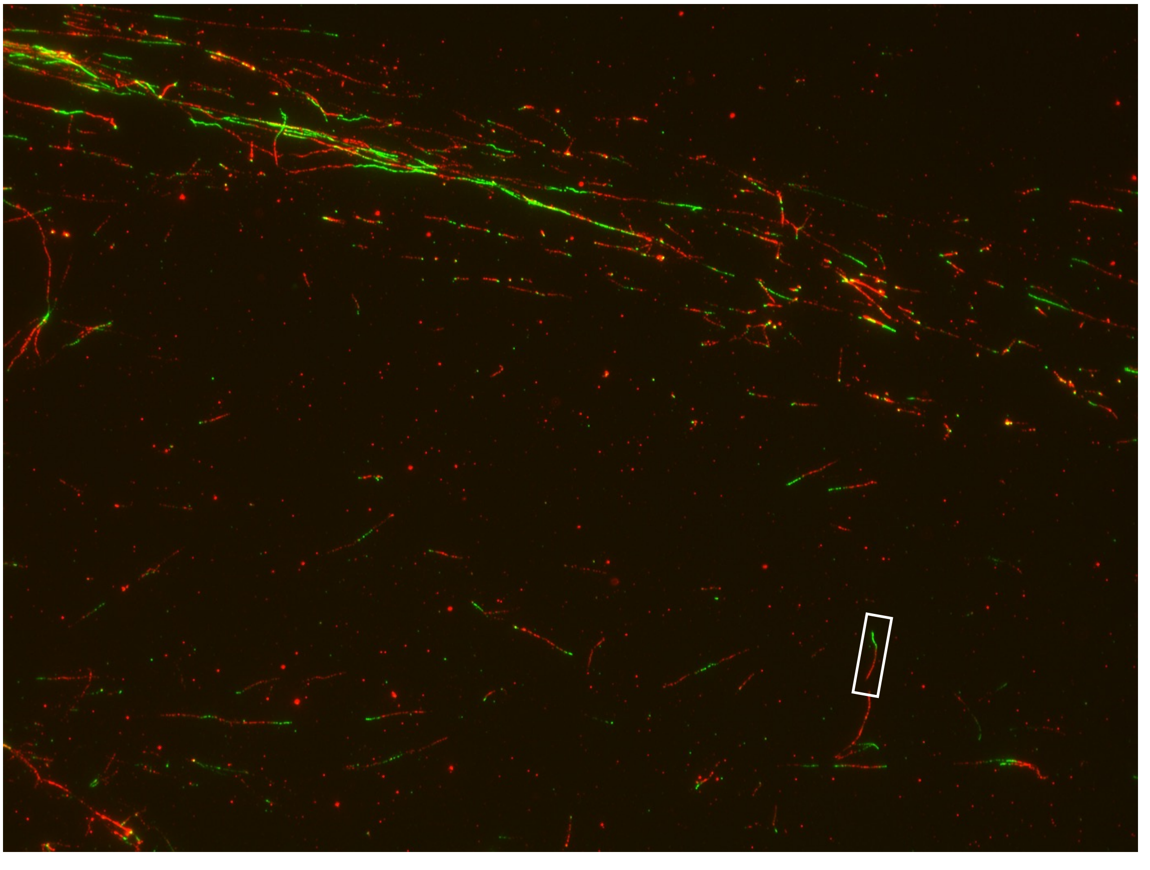

Figure 3c

Unstitched images

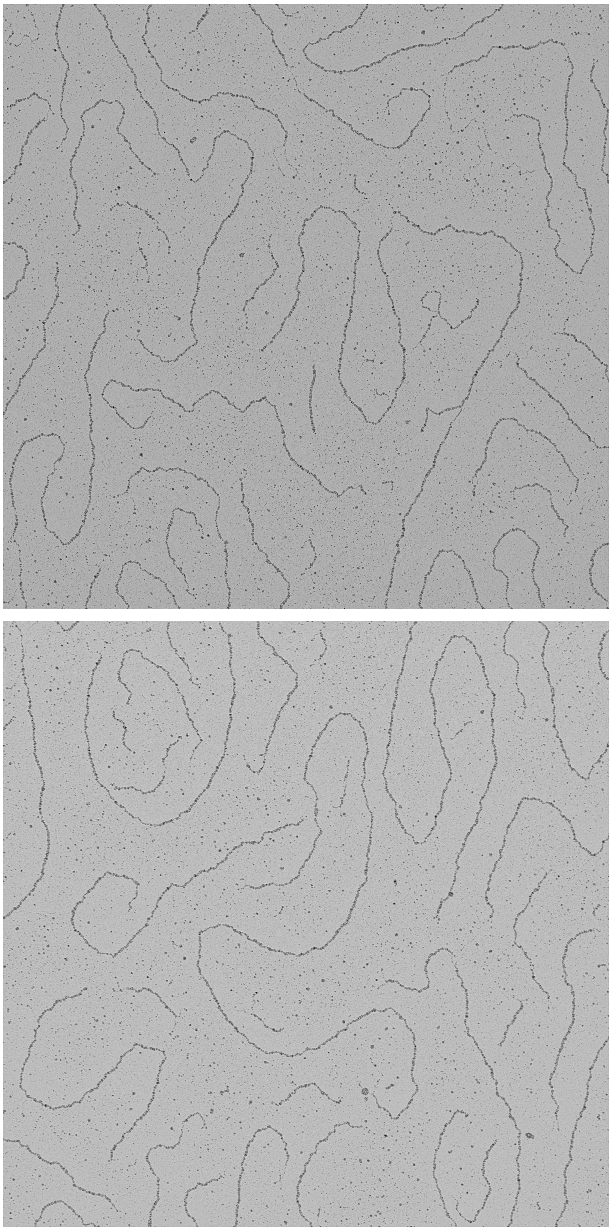

Stitched image

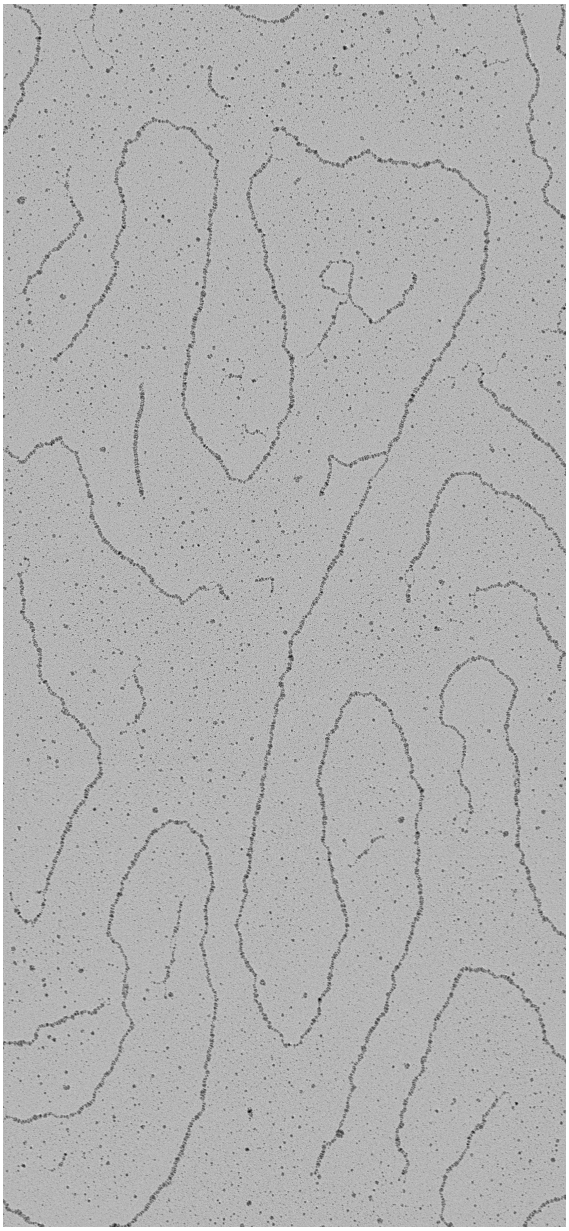

Figure 3g

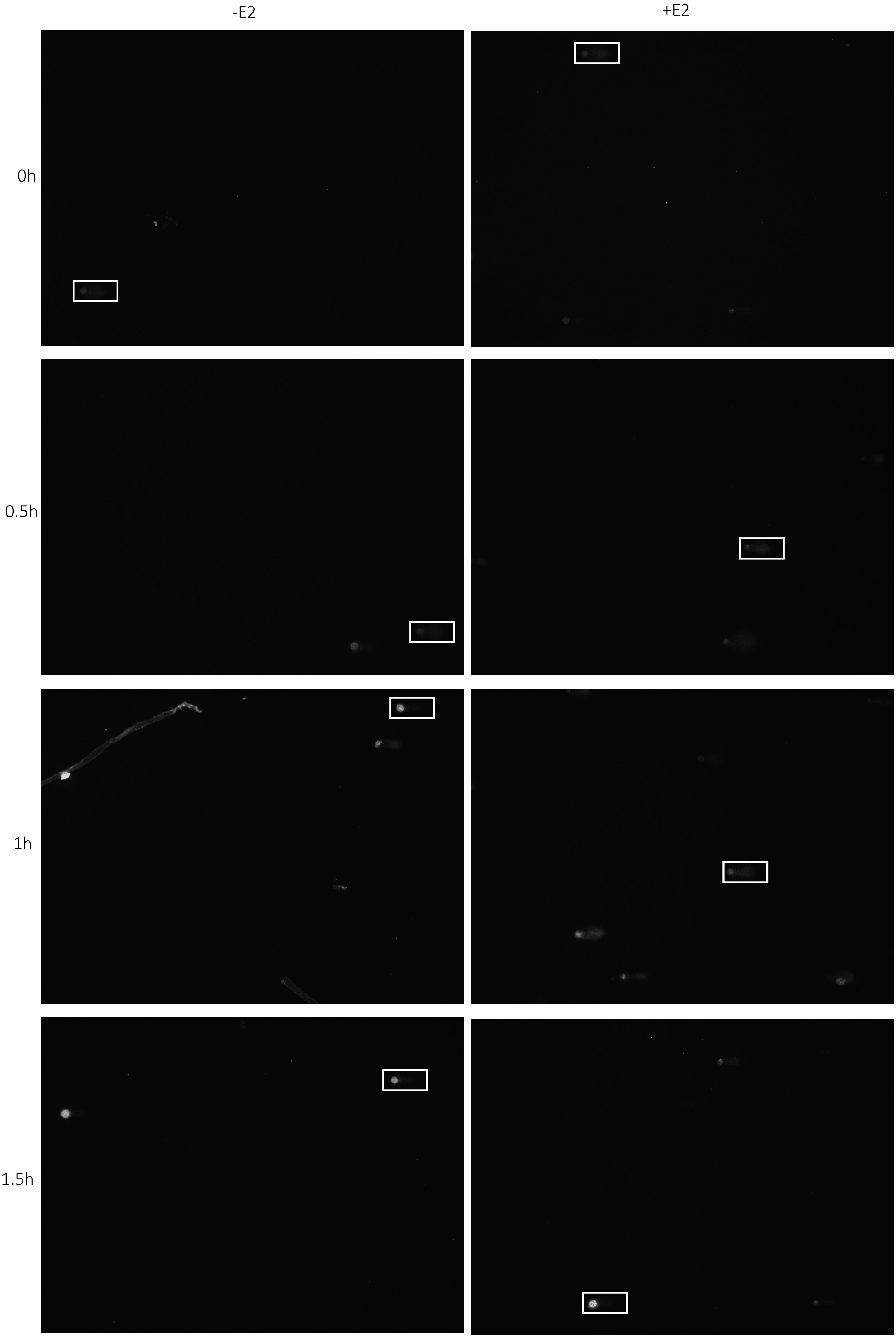

Figure 3h

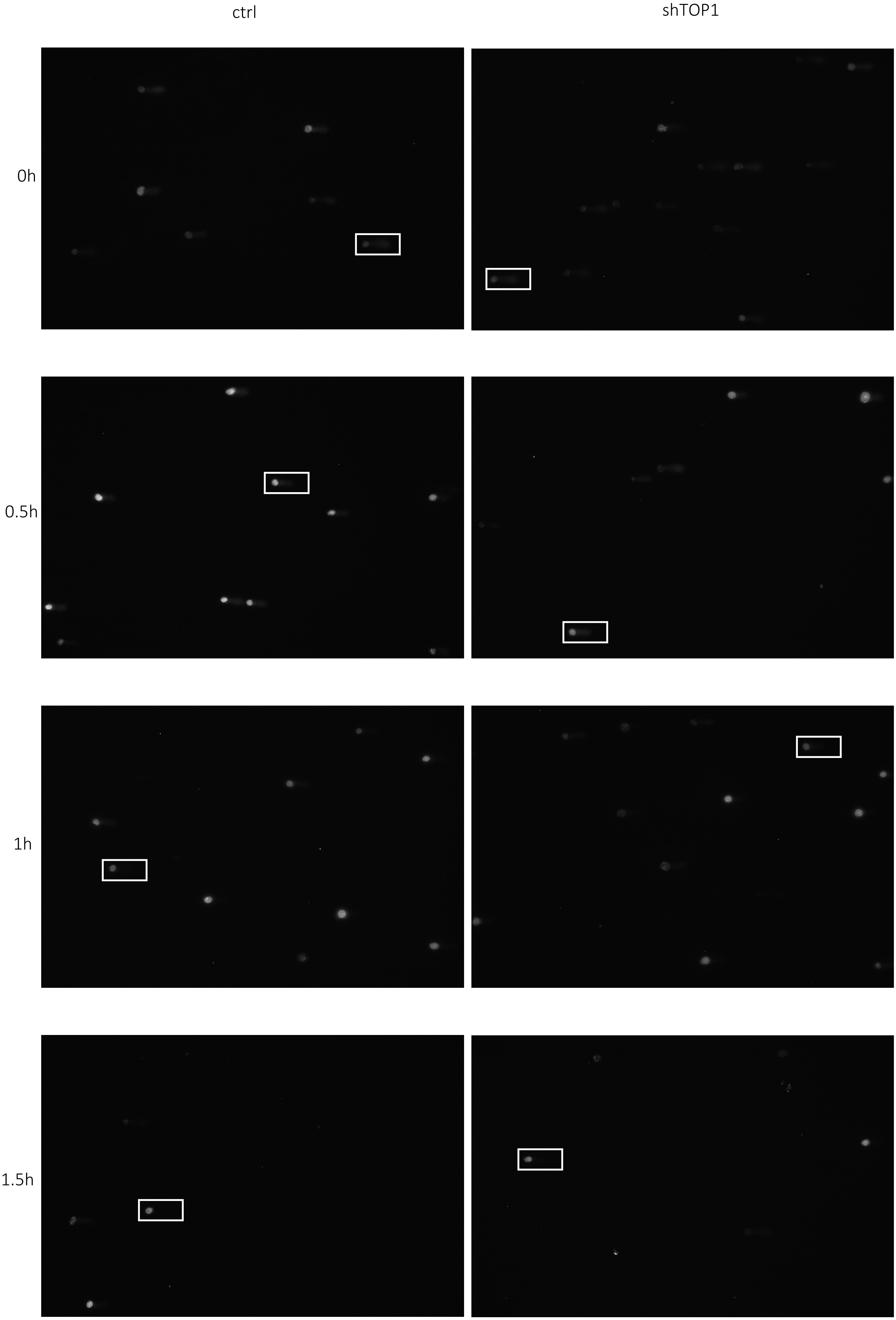

Figure 3i

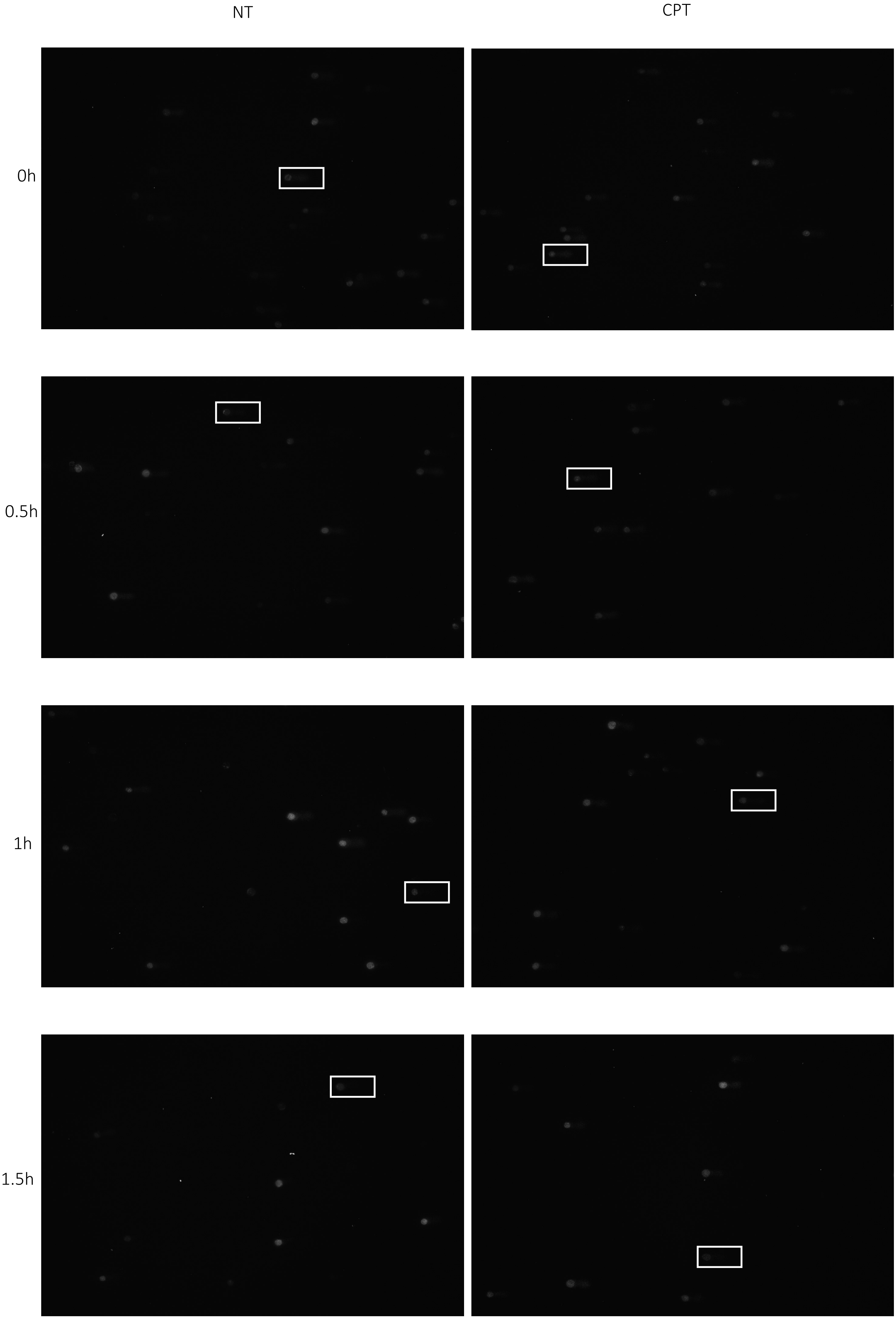

Supplement: Source Data Fig. 3 — Unprocessed representative images. [file 41594_2023_928_MOESM6_ESM.pdf]
