# Supplementary material for: Direct visualization of transcription-replication conflicts reveals post-replicative DNA:RNA hybrids
Source: Nat Struct Mol Biol. 2023 Mar 2;30(3):348–59. doi: 10.1038/s41594-023-00928-6 (PMC10023573; doi:10.1038/s41594-023-00928-6)

Extended Data Figure 2c

S9.6 dsDNA

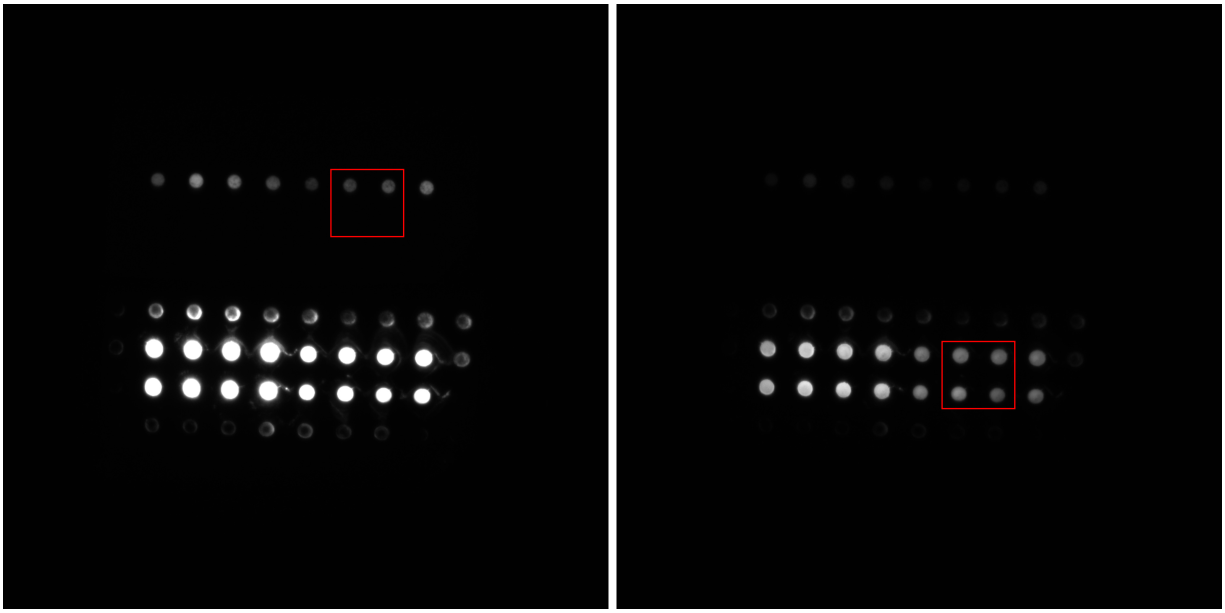

Extended Data Figure 2e

S9.6 Short exposure S9.6 Long exposure dsDNA

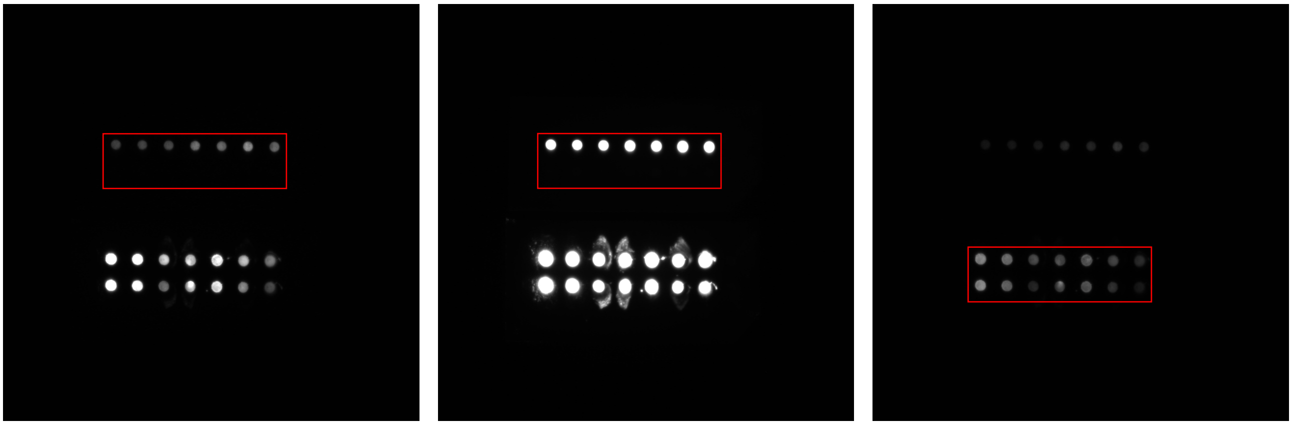

Extended Data Figure 2f

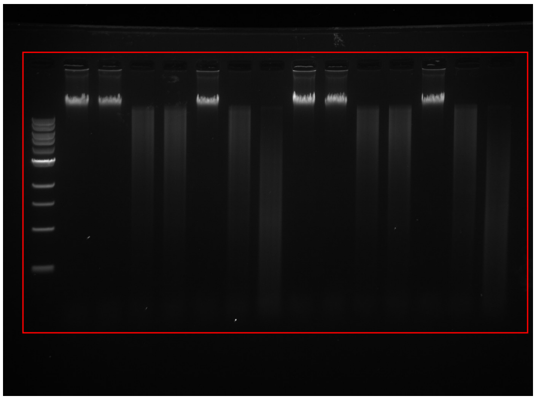

Extended Data Figure 2g

S9.6 Short exposure

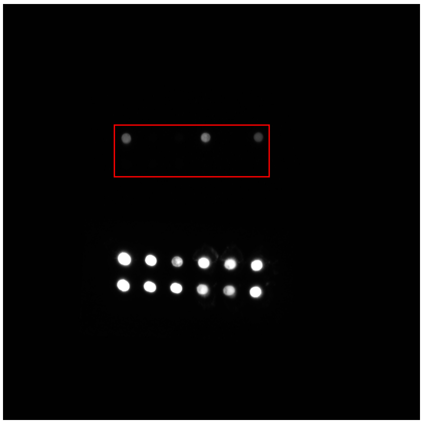

S9.6 Long exposure

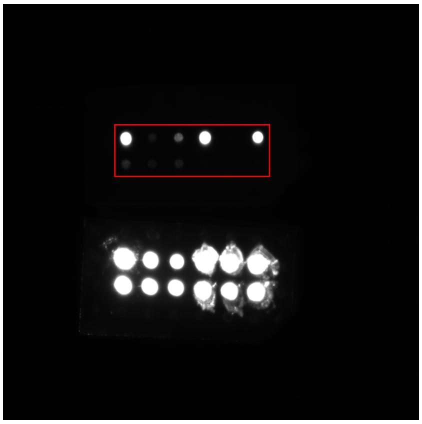

dsDNA

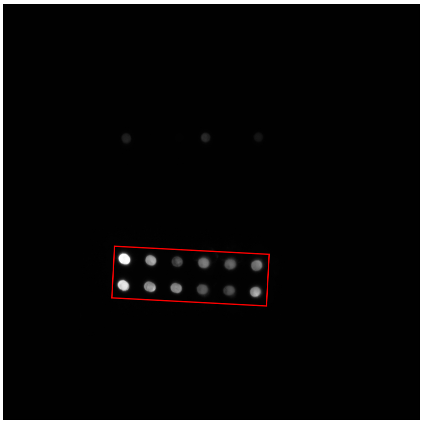

Extended Data Figure 2h

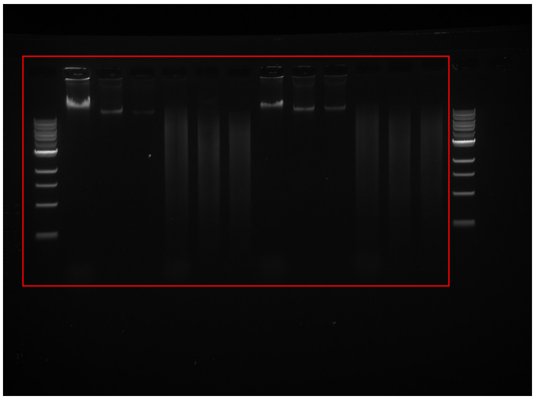

Supplement: Source Data Extended Data Fig. 2 — Unprocessed blots and gels. [file 41594_2023_928_MOESM10_ESM.pdf]
