# Supplementary material for: Direct visualization of transcription-replication conflicts reveals post-replicative DNA:RNA hybrids
Source: Nat Struct Mol Biol. 2023 Mar 2;30(3):348–59. doi: 10.1038/s41594-023-00928-6 (PMC10023573; doi:10.1038/s41594-023-00928-6)

Figure 2b

S9.6

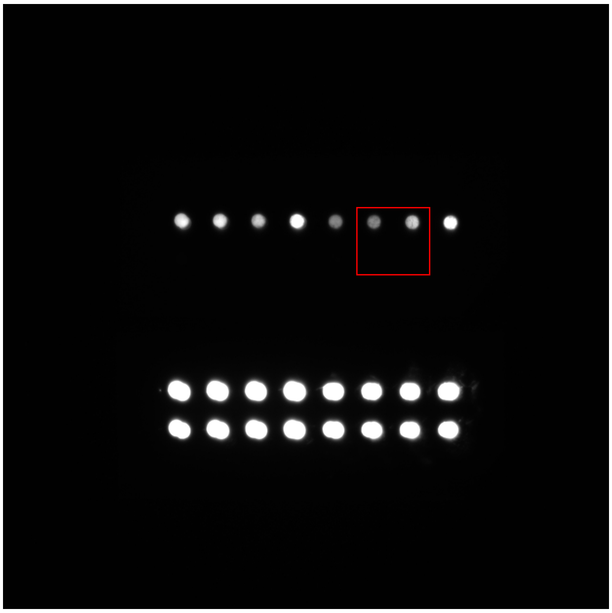

dsDNA

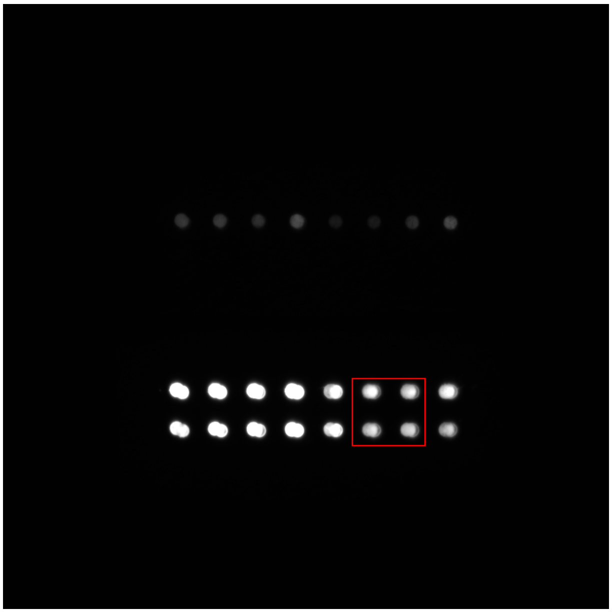

Figure 2d

Raw image | Stitched

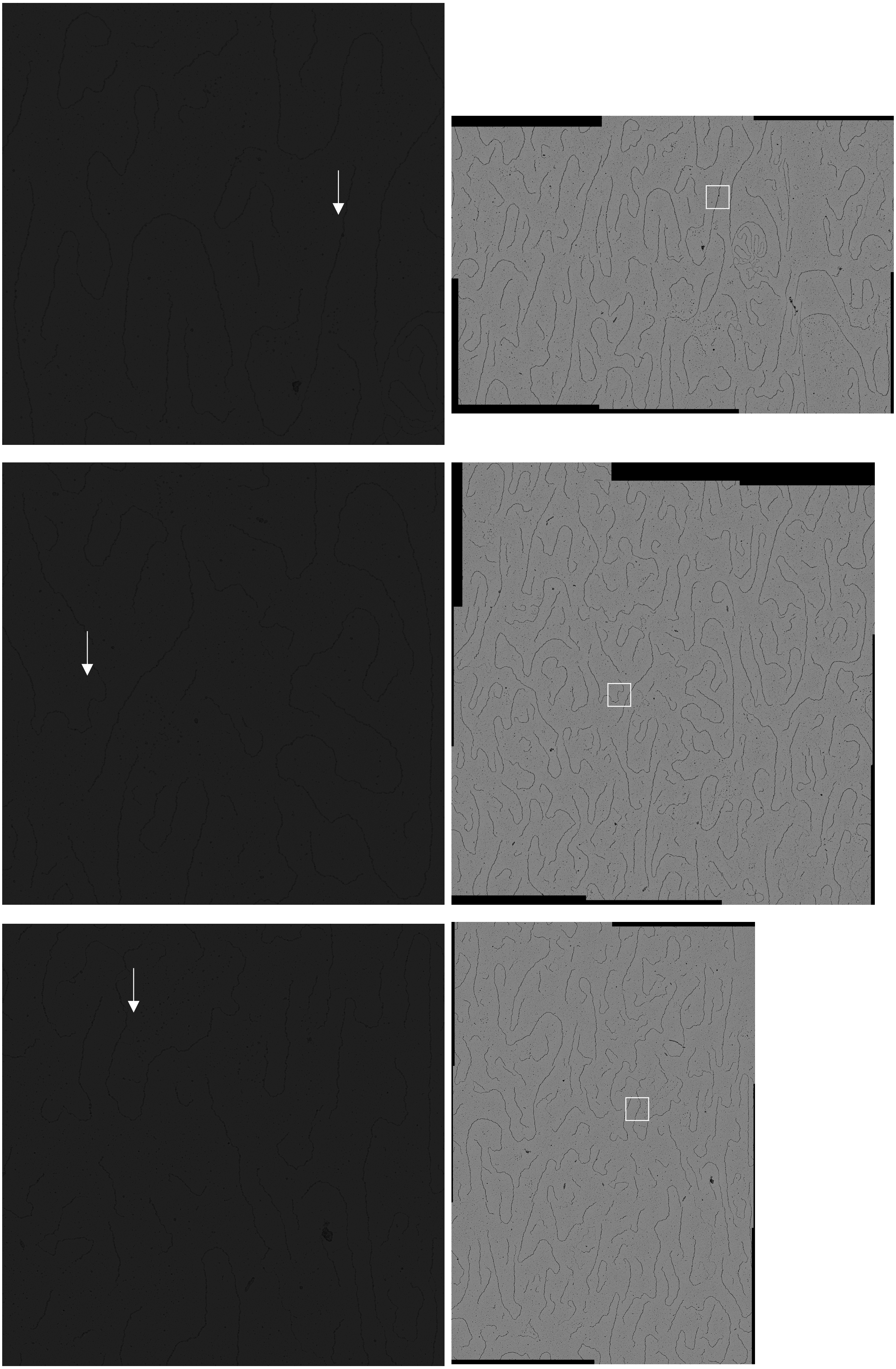

Figure 2d

Raw image | Stitched

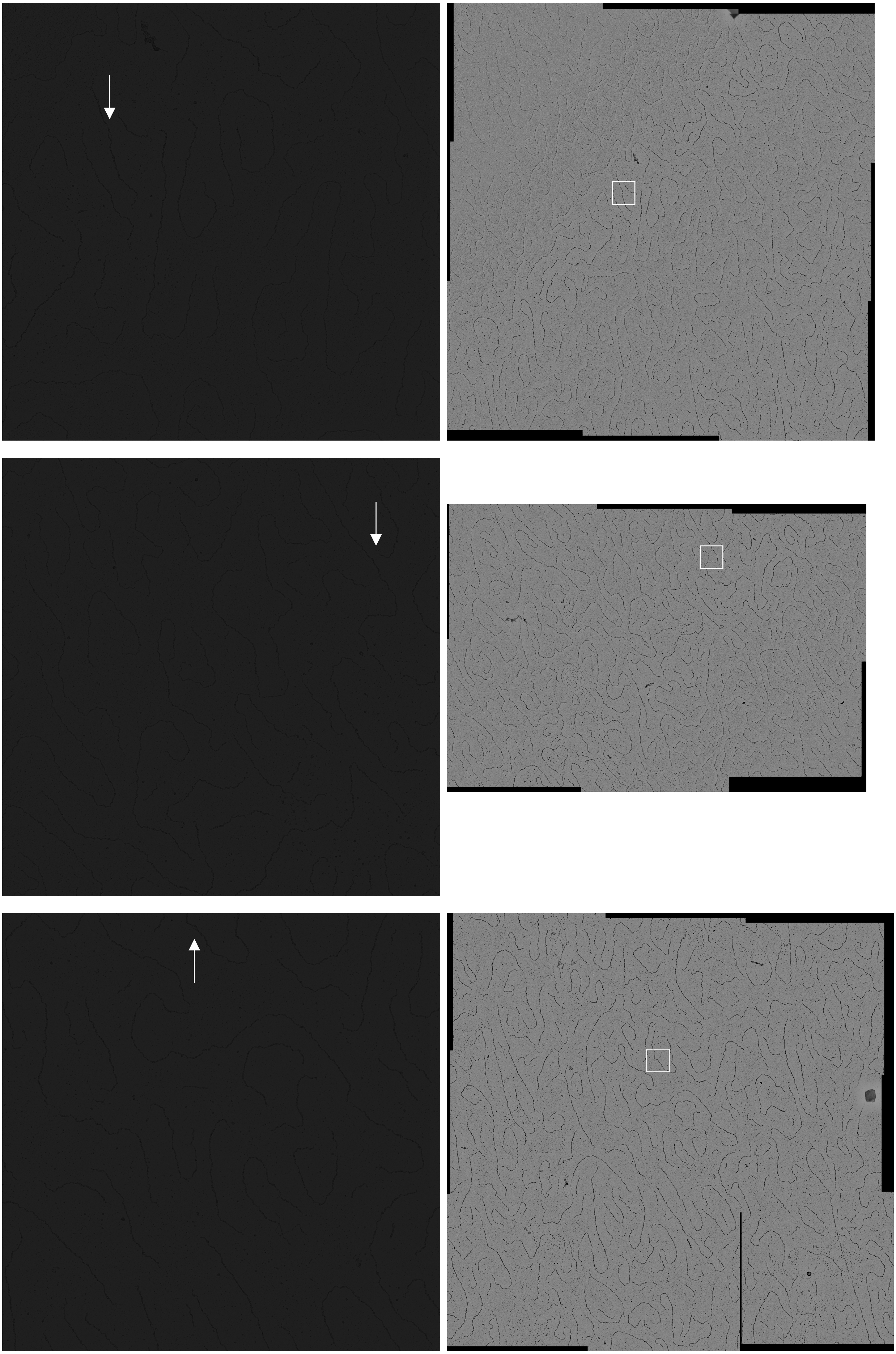

Supplement: Source Data Fig. 2 — Unprocessed blots and representative images. [file 41594_2023_928_MOESM5_ESM.pdf]
