# Supplementary material for: Direct visualization of transcription-replication conflicts reveals post-replicative DNA:RNA hybrids
Source: Nat Struct Mol Biol. 2023 Mar 2;30(3):348–59. doi: 10.1038/s41594-023-00928-6 (PMC10023573; doi:10.1038/s41594-023-00928-6)

Extended Data Figure 3a

Raw Image Stitched image

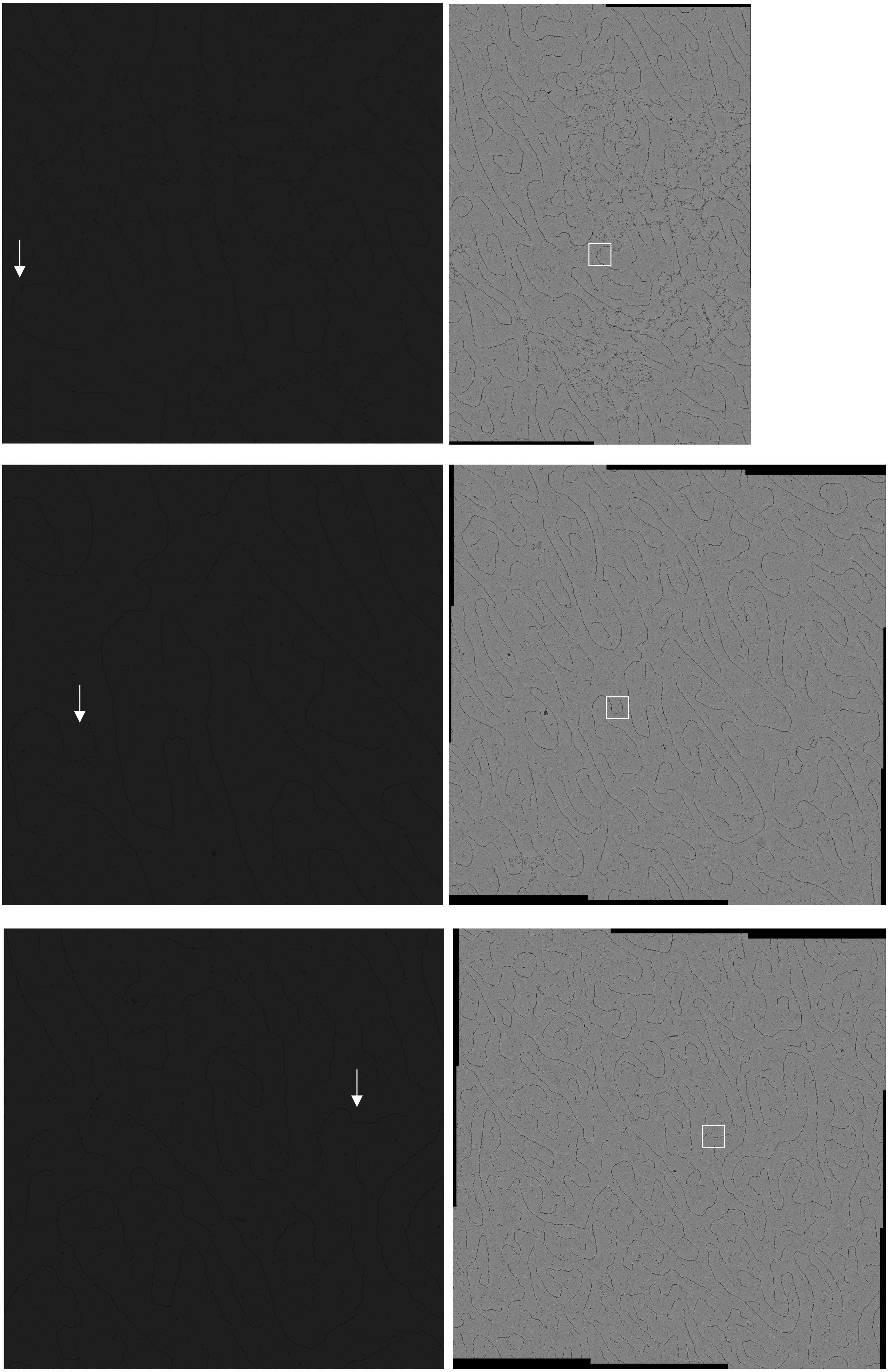

Extended Data Figure 3a

Raw Image

Stitched image

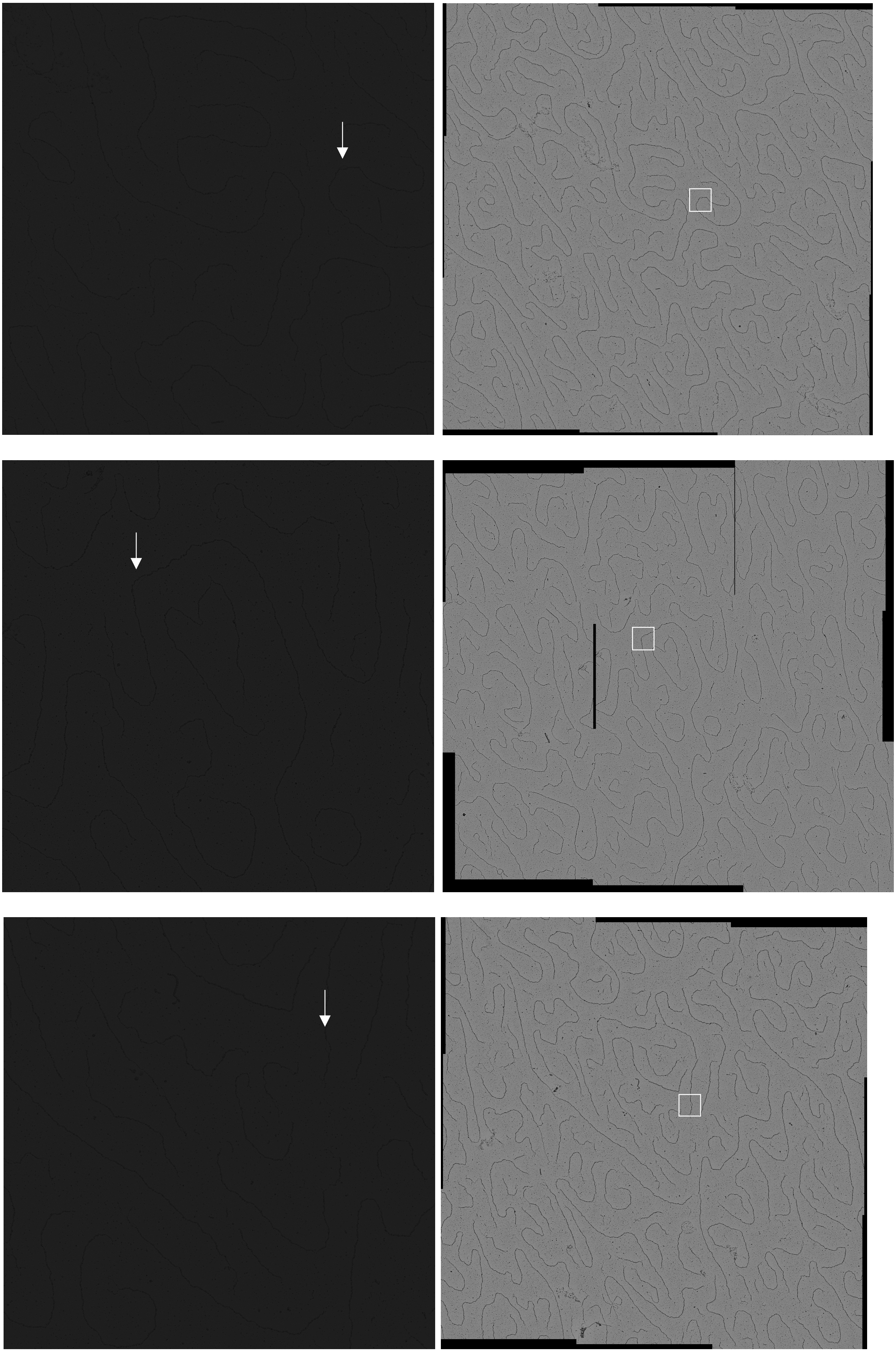

Extended Data Figure 3a

Raw Image | Stitched image

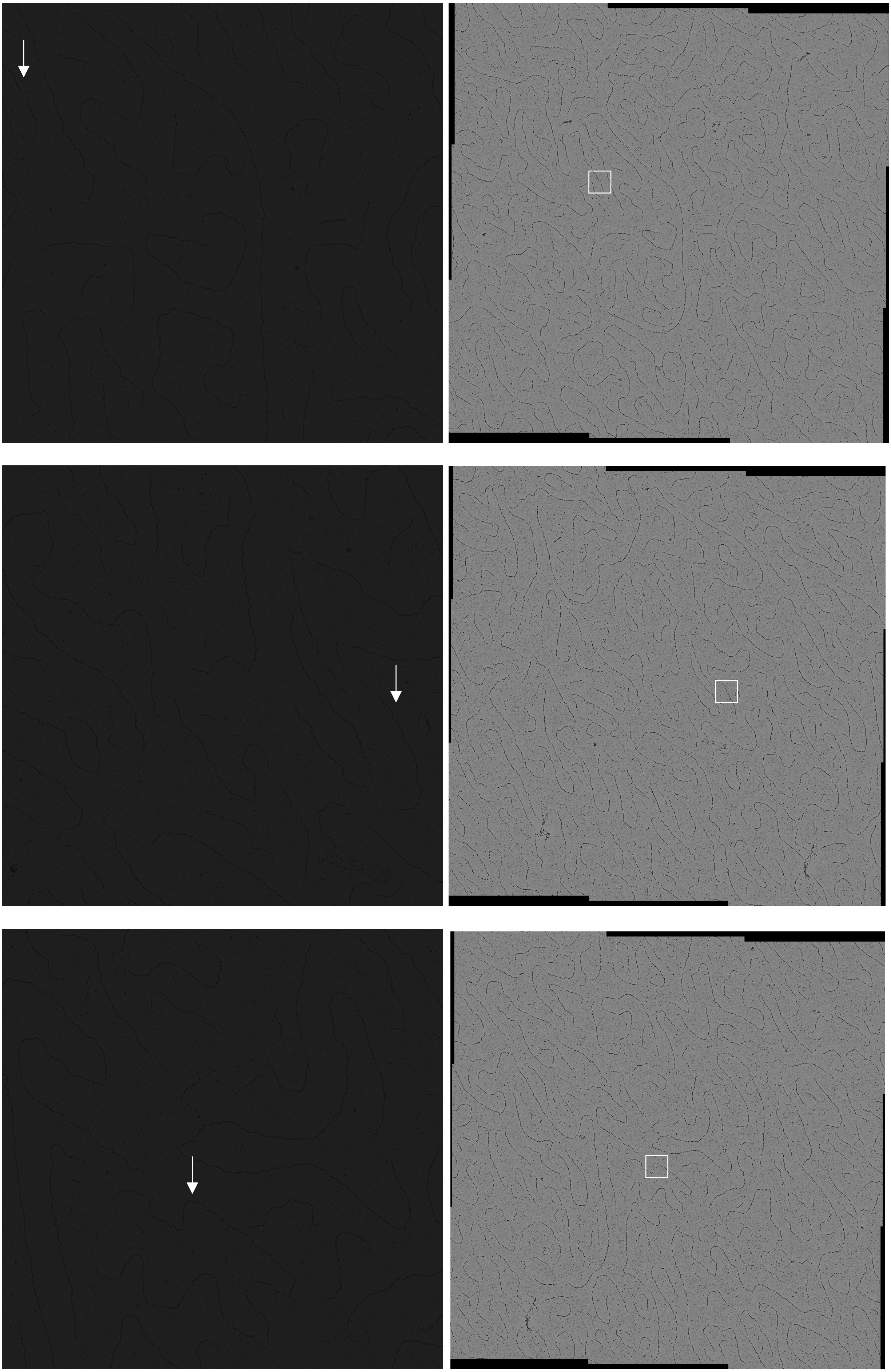

Supplement: Source Data Extended Data Fig. 3 — Unprocessed representative images. [file 41594_2023_928_MOESM11_ESM.pdf]
